# BRCA1-BARD1 associate with the synaptonemal complex and pro-crossover factors and influence RAD-51 dynamics during *Caenorhabditis elegans* meiosis

**DOI:** 10.1371/journal.pgen.1007653

**Published:** 2018-11-01

**Authors:** Eva Janisiw, Maria Rosaria Dello Stritto, Verena Jantsch, Nicola Silva

**Affiliations:** Department of Chromosome Biology, Max F. Perutz Laboratories, University of Vienna, Vienna BioCenter, Vienna, Austria; The University of North Carolina at Chapel Hill, UNITED STATES

## Abstract

During meiosis, the maternal and paternal homologous chromosomes must align along their entire length and recombine to achieve faithful segregation in the gametes. Meiotic recombination is accomplished through the formation of DNA double-strand breaks, a subset of which can mature into crossovers to link the parental homologous chromosomes and promote their segregation. Breast and ovarian cancer susceptibility protein BRCA1 and its heterodimeric partner BARD1 play a pivotal role in DNA repair in mitotic cells; however, their functions in gametogenesis are less well understood. Here we show that localization of BRC-1 and BRD-1 (*Caenorhabditis elegans* orthologues of BRCA1 and BARD1) is dynamic during meiotic prophase I; they ultimately becoming concentrated at regions surrounding the presumptive crossover sites, co-localizing with the pro-crossover factors COSA-1, MSH-5 and ZHP-3. The synaptonemal complex and PLK-2 activity are essential for recruitment of BRC-1 to chromosomes and its subsequent redistribution towards the short arm of the bivalent. BRC-1 and BRD-1 form *in vivo* complexes with the synaptonemal complex component SYP-3 and the crossover-promoting factor MSH-5. Furthermore, BRC-1 is essential for efficient stage-specific recruitment/stabilization of the RAD-51 recombinase to DNA damage sites when synapsis is impaired and upon induction of exogenous damage. Taken together, our data provide new insights into the localization and meiotic function of the BRC-1–BRD-1 complex and highlight its essential role in DNA double-strand break repair during gametogenesis.

## Author summary

Sexually reproducing species rely on meiosis to transmit their genetic information across generations. Parental chromosomes (homologues) undergo many distinctive processes in their complex journey from attachment to segregation. The physiological induction of DNA double strand breaks is crucial for promoting correct chromosome segregation: they are needed to activate the DNA repair machinery responsible for creating physical connections, or crossovers (COs), between the homologues. In turn, crossovers promote the accurate segregation of the chromosomes in daughter cells. The BRCA1–BARD1 complex has a pivotal role during DNA repair in somatic cells and is exclusively located on unaligned chromosomal regions during mammalian meiosis. We show that in *Caenorhabditis elegans*, BRCA1 and BARD1 localize to chromosomes at all stages of meiotic prophase I and are enriched at presumptive crossover sites. We found that BRCA1 promotes DNA loading of the repair factor RAD-51 in specific mutant backgrounds and upon exogenous damage induction. Our data provide evidence for a direct physical association between BRCA1-BARD1 and pro-crossover factors (including the synaptonemal complex) and identify an important role for BRCA1 in stimulating meiotic DNA repair. Further studies are necessary to identify the substrates acted upon by the BRCA1–BARD1 complex to maintain genome stability in the gametes.

## Introduction

The genetic information encoded by DNA must be accurately copied and transmitted from one generation to the next. In somatic cells, DNA is duplicated and equally partitioned into daughter cells via mitosis, whereas in germ cells, which give rise to gametes, chromosome segregation relies on meiosis, a specialized cell division mechanism which produces haploid cells from diploid progenitors. Meiosis requires a unique programme of finely regulated events before cell division to accomplish faithful chromosome segregation. Cognate paternal and maternal chromosomes (homologous chromosomes) find each other (homologous pairing) and then fully align; the interaction is stabilized by formation of the synaptonemal complex (SC). Ultimately, exchange of DNA (recombination) between the homologues chromosomes establishes physical connections, which are essential for faithful segregation [1, 2].

The *Caenorhabditis elegans* gonad is a powerful system for studying chromosomes during both mitosis and meiosis because of the cytological accessibility and the spatio-temporal organization of nuclei into all prophase I stages [3]. Morphological changes to chromosomes mark the engagement of key steps in meiotic progression. At meiotic onset, chromatin adopts a clustered, “half-moon” shape, reflecting chromosome movement and reorganization [4–6]. This structure marks the transition zone (corresponding to the leptotene–zygotene stages). Once homologues are aligned, a tripartite proteinaceous structure called synaptonemal complex (SC) is formed between each homologue pair to allow genetic exchange during CO-dependent DNA repair [1, 2, 7–10]. DNA recombination is initiated by the deliberate induction of DNA double-strand breaks (DSBs) by the topoisomerase II-like enzyme, SPO-11 [11, 12]. In all species, the number of DSBs largely exceeds the final number of COs, suggesting that many DSBs are repaired via pathways such as inter-sister repair (IS) or synthesis-dependent strand annealing [13]. In *C*. *elegans*, most chromosomes receive one CO during meiosis [14], and this depends on the function of many proteins, including the MSH-4/MSH-5 heterodimer (orthologues of the yeast and mammalian MutSγ complex components, MSH4/MSH5) [15–18], the cyclin COSA-1 (orthologue of mammalian CNTD1) [19, 20] and the E3 SUMO-ligase ZHP-3 (orthologue of yeast Zip3) [21–23]. CO formation is abolished in absence of DSBs (e.g. in *spo-11* mutants) or synapsis; however, unlike in other model systems, lack of DNA breaks does not prevent SC formation in *C*. *elegans* [7, 11]. Meiotic DSB repair also relies on RAD-51-mediated repair in *C*. *elegans* [24, 25]: the RAD-51 recombinase localizes to discrete chromatin-associated foci starting in the transition zone and peaking in early pachytene; RAD-51 disengages from DNA in mid-pachytene [7]. Markers of aberrant RAD-51 loading, such as increased foci number and/or extended accumulation, are *bona fide* indicators of defective DSB processing and recombination. CO induction triggers reorganization of the SC components into distinct domains on bivalents (pairs of homologous chromosomes held together by a chiasma): the central elements are confined to the short arm (containing the CO) and the axial elements can be recruited at both the long and the short arm (i.e. HTP-3 and HIM-3) or specifically targeted to the long arm (HTP-1/-2 and LAB-1) [26–30]. This reorganization is particularly evident during diplotene, at which stage bivalents progressively condense and appear as six DAPI-stained bodies in diakinesis nuclei, which are a read-out for the successful execution of prophase I events (aberrant structures include achiasmatic chromosomes (univalents) or fused/fragmented chromatin masses [11, 16, 31]).

The breast and ovarian cancer susceptibility protein BRCA1 and its obligate heterodimeric partner BARD1 form an E3 ubiquitin ligase module (the BCD complex), the functions of which have been extensively studied in mammalian mitotic cells [32]. In this system, it has been shown that the BRCA1–BARD1 heterodimer promotes homologous recombination (HR) during the S–G2 stages, by both favouring extended DNA break resection and preventing the non-homologous end joining (NHEJ)-promoting factor 53BP1 [33] from binding to the site of ongoing DNA repair. Moreover, the activity of the BCD complex also enhances BRCA2 and RAD51 loading at DNA damage sites to elicit accurate DNA repair [32]. *BRCA1*-null mutants are embryonic lethal in mammals, thus hindering the study of this factor in gametogenesis [34–44]. Mouse mutants containing hypomorphic and gain-of-function *BRCA1* alleles show increased apoptotic cell death during spermatogenesis, as well as reduced loading of the pro-CO factor MSH4 and a severe delay in MLH1 focus formation during oogenesis [45]. *C*. *elegans brc-1/BRCA1* and *brd-1/BARD1* mutants are viable and fertile, albeit with increased DNA damage-dependent apoptosis during oogenesis and SPO-11-dependent accumulation of RAD-51 foci, suggesting a defect in processing meiotic recombination intermediates [46, 47]. Importantly, blocking *brc-1-brd-1* function in CO-defective mutants leads to the formation of aberrant chromatin bodies in diakinesis nuclei, underscoring the importance of BRC-1 in the IS repair pathway [47].

Here we report that in the *C*. *elegans* germline, unlike in mammalian systems [48, 49], BRC-1 and BRD-1 are abundantly expressed throughout meiotic prophase I and display a dynamic localization pattern in germ cells, switching from nucleoplasmic expression in early meiotic stages to SC association in pachynema, where they become progressively enriched at the short arm of the bivalent. We provide *in vivo* evidence that both BRC-1 and BRD-1 form complex(es) with both MSH-5 and the SC central element, SYP-3. Localization of BRC-1 and BRD-1 in germ cells is differently regulated by synapsis and CO formation. Finally, we show that the BCD complex promotes stage-specific RAD-51 loading when SC formation is impaired and upon exogenous DNA damage induction. Similar findings are reported by Li and colleagues in the accompanying manuscript. Taken together, our data highlight multiple functions of the BRC-1–BRD-1 heterodimer during gametogenesis.

## Results

### BRC-1 and BRD-1 engage in a mutually dependent, highly dynamic loading in the germline

**Fig 1:**
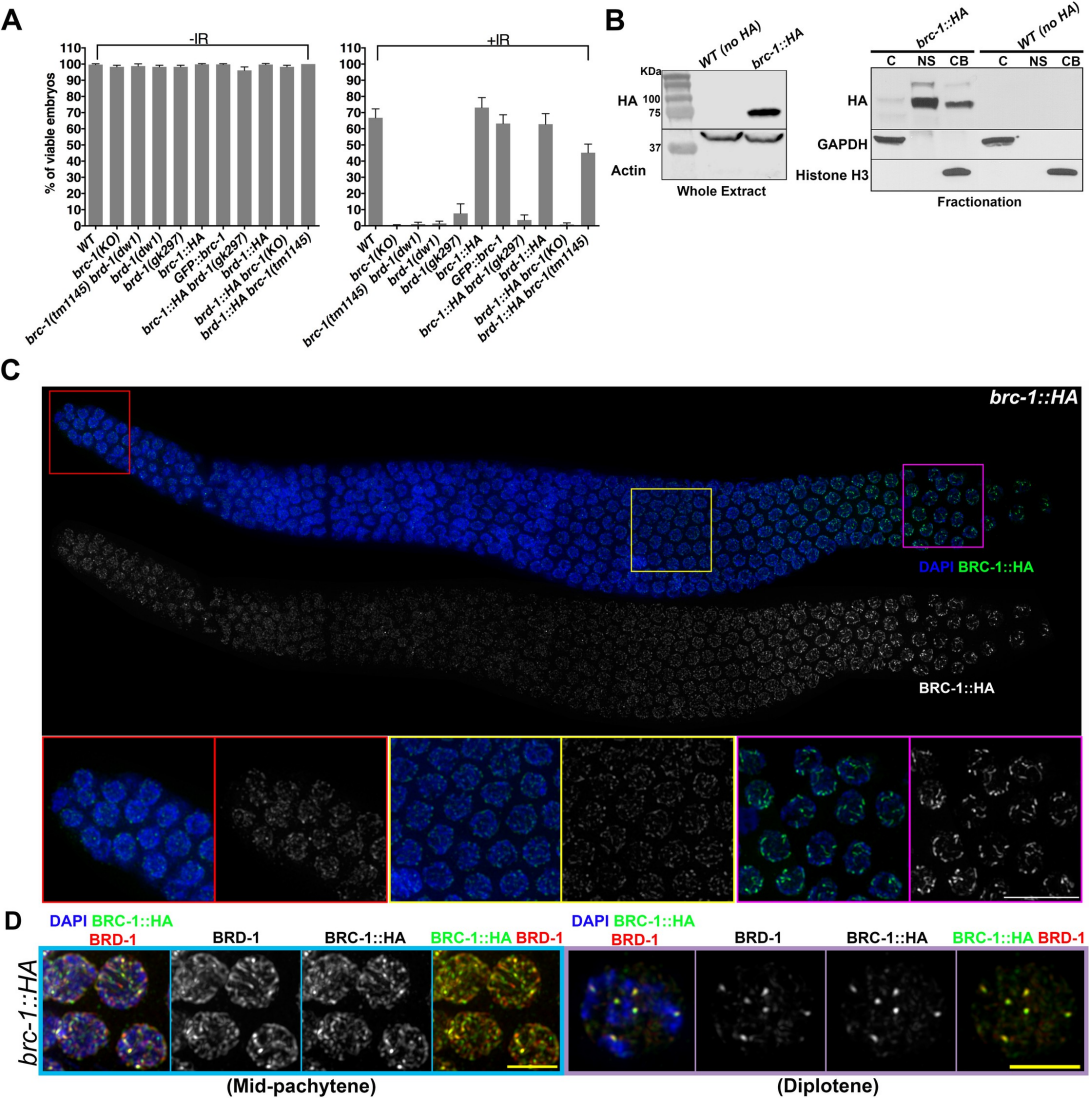
BRC-1–BRD-1 localization during gametogenesis. (A) Embryonic viability levels in various mutant and tagged genetic backgrounds. Functionality of fusion proteins was assessed by exposing worms to IR and then scoring the hatching rate of embryos laid in the following 24 hours. Error bars indicate standard deviation. The number of animal analyzed was: WT (0 Gy, n = 11, 75 Gy, n = 27), *brc-1(KO)* (0 Gy, n = 5, 75 Gy, n = 13), *brc-1(tm1145) brd-1(dw1)* (0 Gy, n = 12, 75 Gy, n = 18), *brd-1(dw1)* (0 Gy, n = 5, 75 Gy, n = 10), *brd-1(gk297)* (0 Gy, n = 5, 75 Gy, n = 19), *brc-1*::*HA* (0 Gy, n = 10, 75 Gy, n = 12), *GFP*::*brc-1* (0 Gy, n = 5, 75 Gy, n = 14), *brc-1*::*HA brd-1(gk297*) (0 Gy, n = 5, 75 Gy, n = 10), *brd-1*::*HA* (0 Gy, n = 5, 75 Gy, n = 11), *brd-1*::*HA brc-1(KO)* (0 Gy, n = 5, 75 Gy, n = 13), *brd-1*::*HA brc-1(tm1145)* (0 Gy, n = 5, 75 Gy, n = 16). (B) Left: western blot analysis using an anti-HA antibody to monitor BRC-1::HA expression in whole-cell extracts. WT (N2) worms were used as negative control to test specificity of anti-HA antibody. Actin was the loading control. BRC-1::HA is predicted to run at 70KDa, which is consistent with the specific band detected slightly below the 75KDa band in the ladder. Right: protein fractionation showing BRC-1::HA enrichment in the nucleus. Equal amounts of protein were loaded for each fraction. C = cytosol, NS = soluble nuclear pool, CB = chromatin-bound pool. GAPDH and histone H3 were used as loading controls for the cytosolic and chromatin-bound samples, respectively. (C) Top: whole-mount gonad from *brc-1*::*HA* worms dissected and stained with DAPI and anti-HA antibody, showing ubiquitous BRC-::HA expression throughout the germline. Note the progressive enrichment on the SC and short arms of bivalents. Bottom: enlarged images of specific regions of the gonad: mitotic tip (red frame), mid-pachytene (yellow frame) and late pachytene/diplotene (magenta frame). Scale bar, 10 μm. (D) Mid-pachytene (light blue frame) and diplotene (light purple frame) nuclei stained with DAPI, anti-HA and BRD-1 antibodies display large co-localization of BRC-1::HA and BRD-1. Scale bar, 5μm.

To gain insight into BRC-1 and BRD-1 function during gametogenesis, we analyzed their localization patterns during meiotic prophase I. To this end, we tagged the endogenous *brc-1* locus with a 5´-GFP or a 3´-HA tag and the *brd-1* locus with a 3´-HA tag using a CRISPR/Cas9 approach [50, 51]. BRD-1 detection was performed by employing a previously characterized antibody as well [46, 52]. Functionality of fusion proteins was assessed by exposing the tagged animals to ionizing radiation (IR): as previously reported [46], *brc-1* and *brd-1* mutants were sterile, whereas *GFP*::*brc-1*, *brc-1*::*HA* and *brd-1*::*HA* worms responded to IR in a similar way as wild-type animals, thus proving that the tagged proteins are fully functional (Fig 1A).

Using a recently established method for isolating germline-enriched proteins [53] involving protein fractionation and western blot analysis, we showed that BRC-1::HA is enriched in the nucleus: most was in the soluble nuclear pool fraction and a smaller proportion was chromatin bound (Fig 1B). Interestingly, unlike in whole-cell extracts, BRC-1::HA was detected as a doublet in fractionated samples, suggesting that a less abundant isoform becomes detectable after enrichment with this extraction method or perhaps the presence of a post-translational modification.

Previous reports indicate that during mouse meiosis, BRCA1 localizes to nascent SC elements in leptotene/zygotene stages; in pachytene cells, it is exclusively located at asynapsed region of the XY-sex body during spermatogenesis or on asynapsed chromosomes during oocyte meiosis [48, 49, 54]. In contrast, in the *C*. *elegans* germline both BRC-1 and BRD-1 were expressed in all nuclei during meiotic prophase I (Figs 1C and S1A) and, as expected, largely co-localized (Fig 1D). Next, we sought to investigate whether, as shown in mammals [55], BRC-1 and BRD-1 display loading interdependency in nematodes. BRD-1 was neither detected in *brd-1(gk297)*, proving antibody specificity, nor in the *brc-1(tm1145)* mutants by immunofluorescence (S1B Fig). However, western blot analysis surprisingly revealed complete lack of BRD-1 expression in *brc-1(tm1145)* mutant worms (S1C Fig), which prompted us to investigate the *brd-1* locus in *brc-1(tm1145)* mutant worms. Genotyping for the *brd-1(dw1)* allele [52] revealed the presence of this deletion in the *brc-1(tm1145)* genetic background (S1C Fig), showing that the *DW102* strain, broadly used in the community as the *brc-1(tm1145)* single mutant, contains the *brd-1(dw1)* deletion in addition to the *brc-1(tm1145)* allele, both closely linked on chromosome III. In order to circumvent this, we then generated the *brc-1*::*HA brd-1* and the *brd-1*::*HA brc-1* mutant backgrounds and analyzed HA staining. BRC-1::HA staining was not detectable in *brd-1(gk297)* mutants (S1D Fig), showing that as in mammals, BRD-1 is essential for BRC-1 loading in the *C*. *elegans* germline. However, BRD-1::HA was normally recruited in *brc-1(tm1145)* mutant germlines (S1D Fig). Irradiation experiments showed that *brc-1(tm1145) brd-1*::*HA* worms displayed a response similar to neither WT nor *brd-1* null mutants (Fig 1A), suggesting that this mutation either does not impair BRD-1 loading or that *tm1145* might be a “separation of function” allele of *brc-1*.

To unambiguously clarify loading and functional dependencies between the two members of the BCD complex, we generated a full knock-out of *brc-1* by CRISPR, in which we deleted the entire *brc-1* locus: *brc-1(KO)* animals displayed similar levels of embryonic lethality as *brd-1* nulls upon IR (Fig 1A) and did not show detectable levels of BRD-1::HA or endogenous BRD-1 by immunofluorescence (S1E Fig). Western blot analysis revealed presence of BRD-1::HA protein in *brd-1*::*HA brc-1(KO)* extracts, confirming the loading impairment (S1F Fig). Interestingly, both BRC-1::HA and BRD-1::HA displayed reduced levels in the reciprocal null mutant backgrounds compared to the relative control animals (36.3% BRC-1::HA in *brd-1* mutants and 45.7% BRD-1::HA in *brc-1(KO)* mutants), suggesting that stability of both BRC-1 and BRD-1 is reduced when the BCD complex cannot be assembled, which was similarly observed in mouse models [56]. In conclusion, our data indicate that loading of BRC-1 and BRD-1 is mutually dependent and that activity of the BCD complex relies on functional integrity of both its members.

### The BCD complex converges to the short arm of the bivalent in a PLK-2-dependent manner

**Fig 2:**
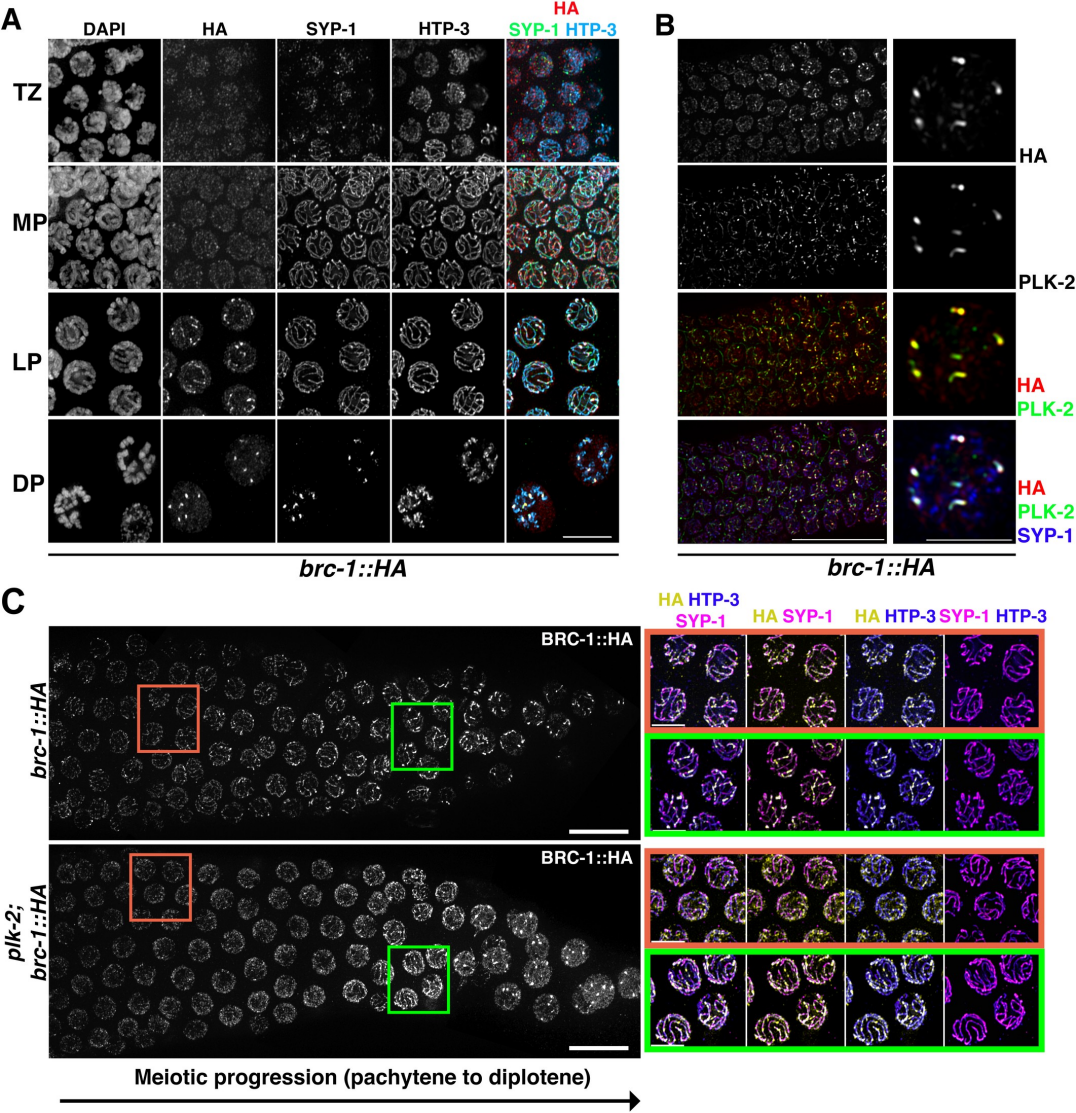
BRC-1 is progressively enriched at the short arms of bivalents in a PLK-2-dependent manner. (A) BRC-1::HA, HTP-3 and SYP-1 localization patterns at different stages of meiotic prophase I. TZ = transition zone, MP = mid-pachynema, LP = late pachynema, DP = diplonema. Scale bar, 5 μm. (B) BRC-1::HA co-staining with anti-PLK-2 and anti-SYP-1 shows BRC-1 recruitment concomitantly with PLK-2 and before SYP-1 to the shorts arm of bivalent. Insets of a representative nucleus from late pachytene/diplotene stage showing full co-localization of BRC-1::HA and PLK-2. Scale bar, 30 μm (left) and 5 μm (right). (C) Left: late pachytene-diplotene transition showing anti HA staining in controls and *plk-2* mutants. Right: insets showing magnified nuclei from picture on the left, from mid-late pachytene (brown rectangle) and late pachytene-diplotene stages (green rectangle) stained for HA (BRC-1), SYP-1 and HTP-3. BRC-1::HA localization along SC and retraction towards the short arm of the bivalent are profoundly impaired by lack of *plk-2* function.

Intriguingly, at the transition between mid- and late- pachytene, BRC-1 and BRD-1 staining switched from a rather diffuse to a discrete linear pattern along the chromosomes; in late pachytene nuclei, BRC-1 and BRD-1 progressively retracted into six short “comet-like” structures (Figs 1C, 1D, S1A and S1B), a specific pattern indicating localization to both CO sites and the short arm of bivalent [7, 8, 21, 22, 57, 58]. To assess whether the BCD complex is indeed recruited to the short arm of the bivalent, we co-stained *brc-1*::*HA* germ lines with antibodies directed against the central element of the SC, SYP-1 [8] and the axial protein HTP-3 [59]. As shown in Fig 2A, BRC-1 co-localized with SYP-1 in late pachytene nuclei, confirming that the BCD complex becomes gradually concentrated at the short arm of the bivalents. Strikingly, BRC-1 enrichment at these regions occurred earlier than observed for SYP-1 (Fig 2A), as six robust SYP-1 stretches were seen only at diplotene stage.

At meiosis onset, the PLK-2 polo-like kinase is enriched at the nuclear envelope attachment sites of chromosome ends, where it promotes homologous pairing and synapsis [60, 61]. In late pachytene, PLK-2 re-locates to discrete domains along the SC, marking local enrichment of recombination factors [62, 63]. PLK-2 redistribution also occurs before SYP-1 redistribution to the short arm of the bivalent and influences the SC structure [62–64]. Given the similar localization kinetics of BRC-1, we co-stained PLK-2 and BRC-1 (Fig 2B) and found that regions enriched for BRC-1 fully overlapped with the PLK-2 staining pattern in late pachytene and diplotene. Thus, we wondered whether BRC-1 localization dynamics required PLK-2 function. Analysis of BRC-1::HA staining in *plk-2* null mutants revealed that BRC-1 association with the SC appeared drastically weakened, although a delayed formation of BRC-1 tracks occurred at the diplotene entry (Fig 2C). Moreover, rather than a gradual re-localization at the short arm as observed in the controls, BRC-1::HA abruptly formed distinct foci and seemingly high levels of mis-localized protein were observed within the nucleus (Fig 2C). We can conclude that the BCD complex is ubiquitously expressed during meiotic prophase I and co-localizes with PLK-2 in pachytene nuclei. Furthermore, PLK-2 promotes progressive enrichment of BRC-1 to the short arm of the bivalent prior to SYP-1 recruitment.

### BRC-1 and BRD-1 co-localize with pro-CO factors MSH-5, ZHP-3 and COSA-1

**Fig 3:**
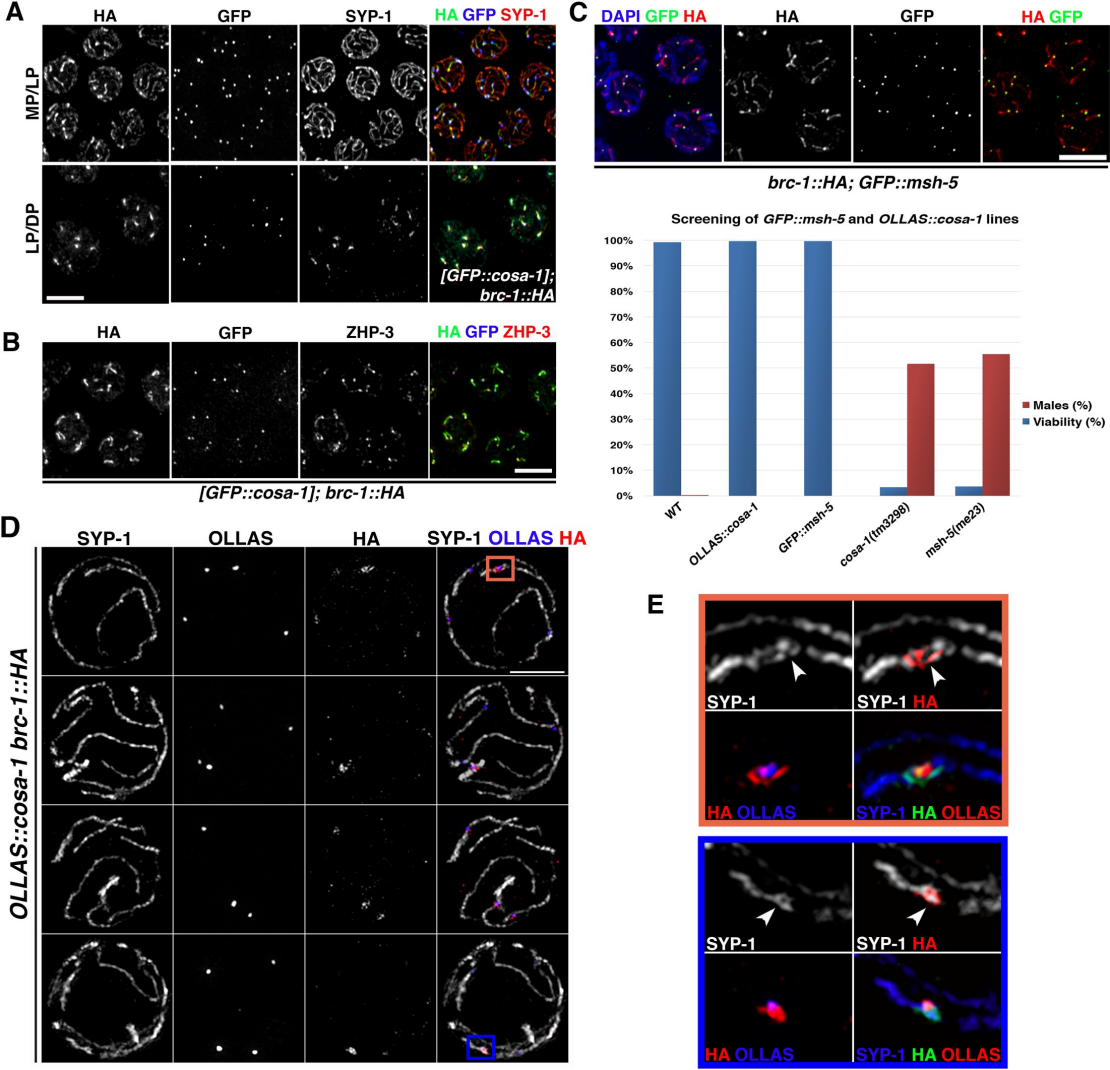
BRC-1 co-localizes with pro-CO factors. (A) BRC-1::HA co-localizes with pro-CO factor COSA-1 in late prophase I. MP/LP = mid-/late pachynema, LP/DP = late pachynema/diplonema. Scale bar, 5 μm. (B) BRC-1::HA co-localizes with ZHP-3 and (C) GFP::MSH-5 in late pachytene nuclei. Scale bar, 5 μm. Bottom: Analysis of embryonic viability and segregation of male progeny shows full functionality of *GFP*::*msh-5* and *OLLAS*::*cosa-1* tagged lines. Embryos scored: WT (1311), *GFP*::*msh-5* (1191), *OLLAS*::*cosa-1* (1098), *msh-5* (971), *cosa-1* (927). (D) Partial projections of nuclei under super-resolution structured illumination microscopy: different examples show BRC-1::HA localization in the region surrounding the COSA-1-labeled CO site. BRC-1::HA forms a nodule-like structure together with SYP-1. (E) Insets showing magnified details from nuclei in (D).

In *C*. *elegans*, formation of inter-homologue COs depends on several proteins, such as the COSA-1/CNTD1 cyclin [20], the MutSγ heterodimer, MSH4/MSH-5 [15, 16] and the ZHP-3 E3 SUMO-ligase [22]. MSH-5 and ZHP-3 are detected at early meiotic stages, with the former accumulating in many foci (these are probably recombination intermediates with both CO and non-CO (NCO) outcomes) and the latter localizing along the SC [20–22]. COSA-1 is prominently detected at mid–late pachytene transition as six foci (one CO for each homologue pair), which also contain MSH-5 and ZHP-3 [20]. Since we observed BRC-1 and BRD-1 recruitment to the short arm of bivalents (chromosome subdomains caused by the formation of CO intermediates [26, 27, 29]), we wondered whether local enrichment of the BCD complex coincides with the regions labeled with pro-CO factors. Comparison of the localization dynamics of GFP::COSA-1 and BRC-1::HA showed that BRC-1 starts to become concentrated concomitantly with enhanced COSA-1 loading in mid-late pachytene nuclei and defines a discrete area which later also contains SYP-1 (Fig 3A). We obtained the same localization pattern by monitoring BRD-1 loading (S2 Fig).

Furthermore, staining with anti-ZHP-3 antibody [21] also revealed full co-localization with BRC-1 (Fig 3B). To evaluate BRC-1 co-localization with MSH-5, we tagged the endogenous *msh-5* locus with a 5′ GFP tag by CRISPR/Cas9. The tagged line was fully functional, as it showed normal levels of fertility (Fig 3C), suggesting that GFP::MSH-5 is competent in promoting CO formation. Similar to ZHP-3 and COSA-1, BRC-1::HA labeled the entire chromosomal region bearing the GFP::MSH-5 foci (Fig 3C). We performed structured illumination microscopy (SIM) to further analyze BRC-1 association with the CO site. For this, we added a 5′ OLLAS-tag to the endogenous *cosa-1* locus [65, 66]. This fully functional line (Fig 3C) was recombined into *brc-1*::*HA* worms and co-stained for OLLAS (COSA-1), BRC-1 and SYP-1. This further confirmed BRC-1 enrichment around COSA-1-labeled CO sites (Fig 3D) and due to the higher resolution provided by the SIM microscopy, we could appreciate in these nuclei that BRC-1 decorates the region of the SC embracing the putative recombination site; thus, it appears to surround, rather than overlapping with, COSA-1 (Fig 3E).

### BRC-1 and BRD-1 physically interact with MSH-5 and SYP-3 *in vivo*

**Fig 4:**
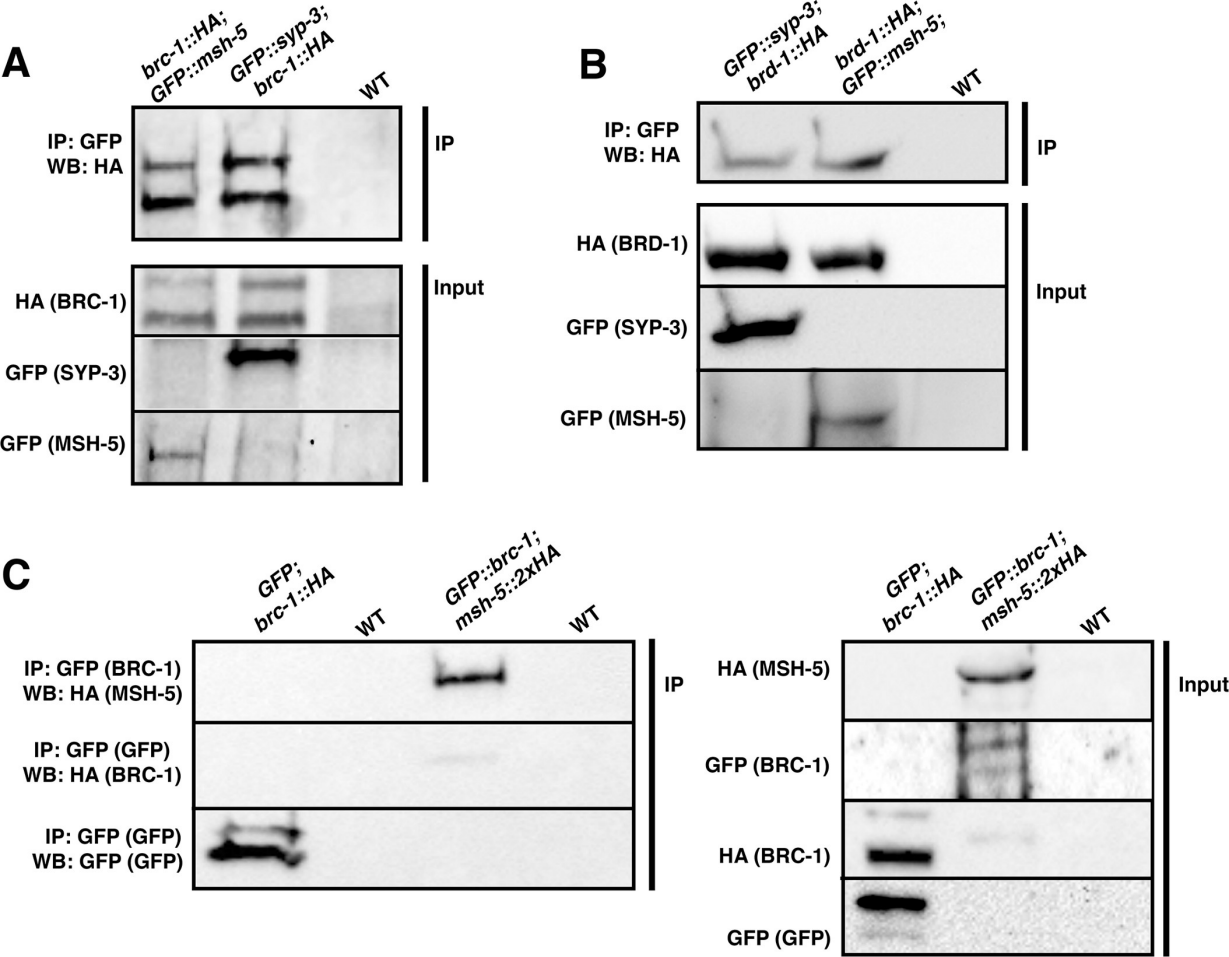
BRC-1 and BRD-1 form complexes with MSH-5 and SYP-3 *in vivo*. (A) Pull-downs of GFP::MSH-5 and GFP::SYP-3 reveals presence in a complex with BRC-1::HA. (B) BRD-1::HA co-immunoprecipitates with GFP::MSH-5 and GFP::SYP-3 as well. (C) Presence in a protein complex of MSH-5 and BRC-1 was further validated by performing reciprocal IPs in which the GFP and HA tags were swapped (*msh-5*::*2xHA* and *GFP*::*brc-1* strains). Non-specific binding of BRC-1 with GFP was ruled out by performing IPs of BRC-1::HA in a strain carrying an integrated GFP with ubiquitous expression (see Methods).

Given their spatial association with both CO factors and the SC, we wondered whether BRC-1 and BRD-1 formed protein complexes with these factors *in vivo*. We performed pull-down experiments using the *brc-1*::*HA; GFP*::*msh-5* strain (Fig 3) and crossed *brc-1*::*HA* into worms expressing a single-copy insertion transgene encoding a largely functional GFP::SYP-3 protein [67]. The same was done to generate *brd-1*::*HA; GFP*::*msh-5* and *GFP*::*syp-3; brd-1*::*HA* strains. Cytosolic, nuclear soluble and chromatin-bound protein fractions [53] were produced from all the above mentioned strains and both nuclear fractions were pooled for immunoprecipitation experiments. Western blot analysis using anti-HA antibodies on GFP pull-downs revealed co-immunoprecipitation of both BRC-1::HA and BRD-1::HA with GFP::MSH-5 and GFP::SYP-3 (Fig 4A and 4B).

Furthermore, we also generated i) a *GFP*::*brc-1; msh-5*::*2xHA* strain to perform reciprocal IPs and assess whether co-IP of BRC-1 and MSH-5 was still occurring upon tag swapping, and ii) a *GFP; brc-1*::*HA* strain in which the GFP was expressed, in order to rule out non-specific binding. MSH-5::2xHA co-immunoprecipitated also with GFP::BRC-1 (Fig 4C), recapitulating, and further validating the result obtained with the *brc-1*::*HA; GFP*::*msh-5* line (Fig 4A). Importantly, no interaction was observed between BRC-1::HA and the GFP alone (Fig 4C), confirming specificity of the interactions (Fig 4A). Therefore, we can conclude that BRC-1 and BRD-1 form complex(es) with both MSH-5 and SYP-3 proteins *in vivo*. These results reveal a previously unknown physical interaction of the BCD complex with pro-CO factors, as well as SC components, highlighting a possible role for BRC-1–BRD-1 at the interface between synapsis and recombination.

### CO establishment triggers redistribution of the BRC-1–BRD-1 complex

**Fig 5:**
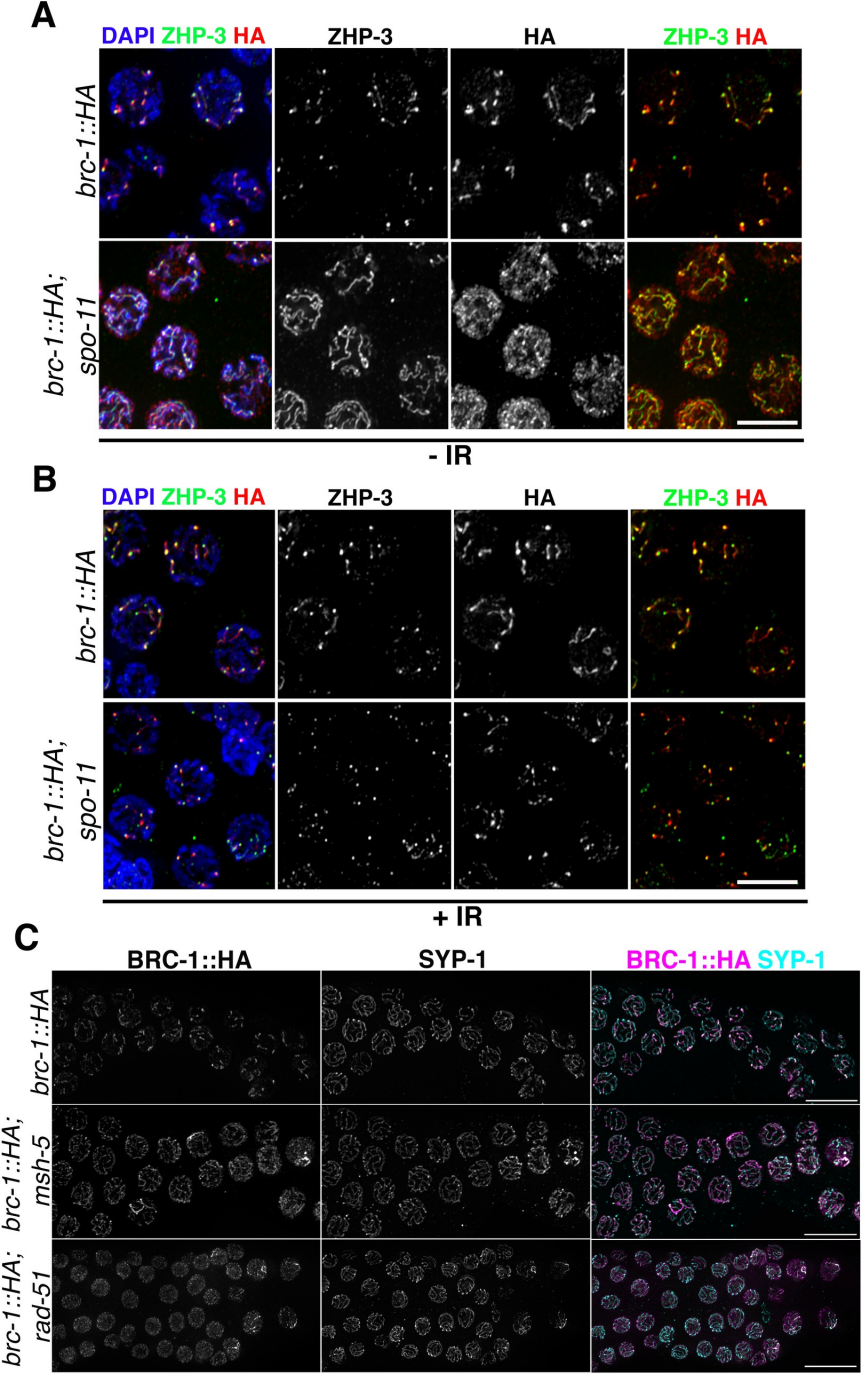
Establishment of chiasmata triggers BRC-1 and BRD-1 redistribution. (A) Late pachytene nuclei in non-irradiated samples show altered BRC-1::HA localization in *spo-11* mutants: BRC-1 and ZHP-3 remain largely localized along the SC without retraction to the short arms of bivalents. Scale bar 5 μm. (B) Ionizing radiation rescues ZHP-3 and BRC-1::HA redistribution in *spo-11* mutants. Scale bar 5 μm (C) In the *msh-5* and *rad-51* mutants, BRC-1::HA accumulates in the SC and it displays interspersed staining respectively. In addition, in *rad-51* mutant germlines, association of BRC-1::HA with the SC is strongly reduced, although still present. Scale bar, 10 μm.

To assess whether BRC-1–BRD-1 redistribution depends on CO establishment, we generated a *brc-1*::*HA*; *spo-11* mutant strain to monitor BRC-1::HA loading in absence of meiotic DSBs, which are essential for inducing CO formation. We found that BRC-1 and ZHP-3 retraction toward the CO site was largely impaired (Fig 5A) although sporadically their redistribution was observed in late pachytene nuclei (S3 Fig), most likely due to formation of spontaneous or pre-meiotic DSBs which are proficient in triggering recruitment of CO-promoting factors and therefore elicit remodelling of the SC components and give rise to a chiasma [20].

Exogenous DSB induction is sufficient to temporarily restore COSA-1 loading and therefore chiasmata formation in *spo-11* mutants [11, 20, 64]. Thus, we investigated whether γ-irradiation could rescue failure in BRC-1 redistribution. We exposed *brc-1*::*HA; spo-11* mutant worms to 20 Gy and analyzed BRC-1 and ZHP-3 loading at 8 hours post-irradiation: at this time point, all late pachytene nuclei in *spo-11* mutants display six COSA-1 foci, suggesting that CO designation is fully rescued [20]. In the irradiated samples, ZHP-3 was retracted towards the CO site and, consistently, BRC-1 also became concentrated on the short arm (Fig 5B). Based on these data, we conclude that BRC-1 and BRD-1 localize to the short arms of bivalents and their reorganization in mid-pachytene nuclei is dependent on CO establishment.

### Recombination and synapsis differently impact on BRC-1 and BRD-1 loading

**Fig 6:**
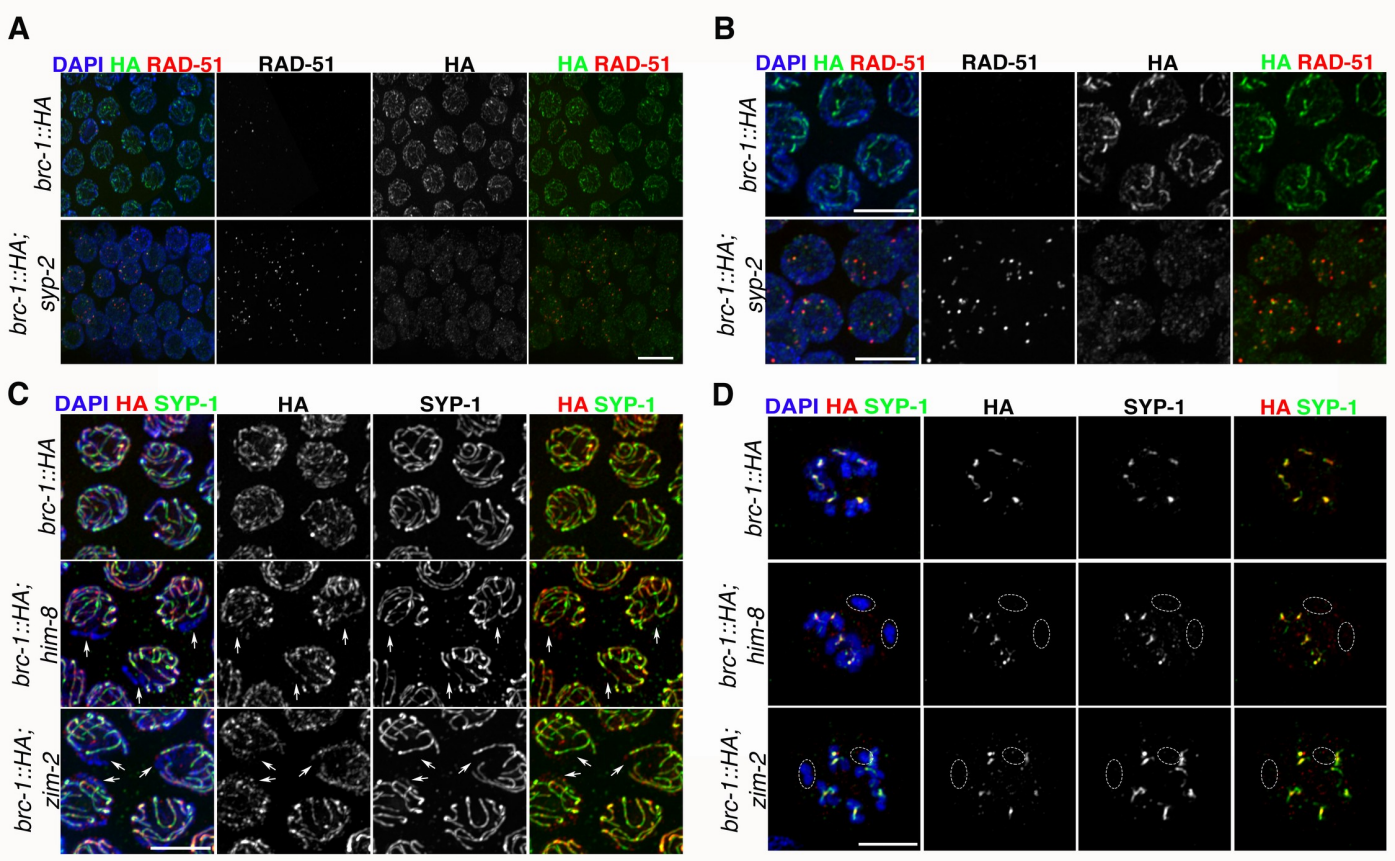
Synapsis differentially regulates BRC-1 localization. (A) Abrogation of synapsis triggers BRC-1::HA accumulation into discrete chromatin-associated foci which co-localize with RAD-51 in late pachytene cells. Scale bar, 5 μm. (B) Insets of nuclei from (A) showing punctate accumulation of BRC-1::HA and co-localization with RAD-51. (C) BRC-1::HA does not accumulate on asynapsed chromosomes X or V in late pachytene nuclei of *him-8* or *zim-2* mutant respectively, but the remaining chromosomes display normal BRC-1::HA loading. Arrows indicate only the most obvious regions of DNA devoid of both SYP-1 and BRC-1::HA. Scale bar, 5 μm. (D) Diplotene nuclei of *him-8* and *zim-2* mutants clearly lack BRC-1::HA on asynapsed univalents (circled). Scale bar, 5 μm.

Given that CO establishment triggers BRC-1–BRD-1 redistribution, we sought to analyze their localization in mutants impaired at different steps of CO formation. As already mentioned, an absence of DSBs leads to a lack of recombination, which largely prevents BRC-1 and BRD-1 retraction to the short arms of bivalents. We therefore asked whether impaired DNA repair by HR, but not by DSB induction, influences BRC-1 and BRD-1 localization. To address this, we crossed *brc-1*::*HA* into the *msh-5* mutant, where conversion of recombination intermediates into mature CO products is prevented [7, 16]. In *msh-5* mutants, BRC-1 accumulated along the SC but retraction was not observed (Fig 5C), similar to the localization pattern observed in *spo-11* (Fig 5A). Then, we analyzed BRC-1::HA staining in *rad-51* mutants, which have normal SC assembly but no homologous DNA repair due to lack of RAD-51-dependent strand displacement and invasion of the homologous chromosome [24, 25]. Interestingly, BRC-1 had a rather punctate staining pattern but despite this, a strong association with SYP-1 in chromosome subdomains was observed in nuclei exiting the pachytene stage (we also observed this in *msh-5* mutants) (Fig 5C). We observed a similar pattern of BRD-1 localization in *com-1* mutants (S4 Fig): here, interfering with DSB resection impairs RAD-51 loading and therefore abolishes CO formation [68]. These results suggest that a lack of COs *per se* impairs redistribution of the BCD complex in late pachytene cells without perturbing loading along the SC. However, in mutants such as *rad-51* that are defective in the early steps of recombination, BRC-1–BRD-1 association with the SC is also dramatically reduced. Next, we sought to analyze whether BRC-1 and BRD-1 loading is regulated by synapsis. We analyzed BRC-1::HA loading under complete and partial absence of SC, as well as in mutants in which synapsis occurs between non-homologous chromosomes. The central portion of the SC is formed by several proteins (SYP-1–4) which are loaded in an interdependent manner; thus, all are necessary to establish synapsis [7, 8, 57, 58]. In the *syp-2* synapsis-null mutant [7], BRC-1::HA had a rather punctate staining pattern throughout meiotic prophase I (Fig 6A). Strikingly, unlike in the wild type, where BRC-1 starts to spread along the SC immediately after the disappearance of RAD-51, in *syp-2* mutants BRC-1 foci remained in close proximity to and co-localized with RAD-51 in mid- and late-pachytene nuclei (Fig 6A and 6B).

In *C*. *elegans*, a family of zinc-finger nuclear proteins connects chromosome-specific ends (i.e. pairing centres) to the nuclear envelope to promote chromosome pairing and synapsis [69, 70]. ZIM-2 and HIM-8 bind to the ends of chromosomes V and X, respectively. Therefore, chromosome V is asynapsed in *zim-2* mutants and chromosome X is asynapsed in *him-8* mutants. We asked whether a partial deficiency in synapsis establishment (affecting only one chromosome pair) also changed BRC-1 loading dynamics. Analysis of BRC-1::HA expression in *him-8* and *zim-2* mutants revealed lack of BRC-1 on unsynapsed chromosomes pairs, despite normal loading along the SC and retraction towards the CO site in the remaining bivalents (Fig 6C and 6D), suggesting that local synapsis defects do not impair global BRC-1 loading. Lastly, we analyzed BRD-1 loading in two mutants with aberrant SC assembly. HTP-1 is a HORMA-domain-containing protein essential to achieve normal levels of pairing and preventing SC assembly between non-homologous chromosomes, while PROM-1 is an F-box protein involved in promoting meiotic entry and homologous pairing. Both *htp-1* and *prom-1* mutants display extensive SYP-1 loading between non-homologous chromosomes as well as asynapsed chromosome regions; consequentially, chiasmata formation is severely impaired [26, 71]. Remarkably, the degree of BRD-1 co-localization with SYP-1 was extremely reduced in both *htp-1* and *prom-1* mutants, with most BRD-1 detected as bright agglomerates within the nucleus (S5A Fig). The same localization pattern was observed for BRC-1::HA in *htp-1* mutants (S5B Fig). Thus, we conclude that BRC-1 and BRD-1 redistribution during meiotic progression requires CO establishment, is tightly regulated by the SC and does not follow SC installation between non-homologous chromosomes.

### The BCD complex promotes efficient processing of recombination intermediates

**Fig 7:**
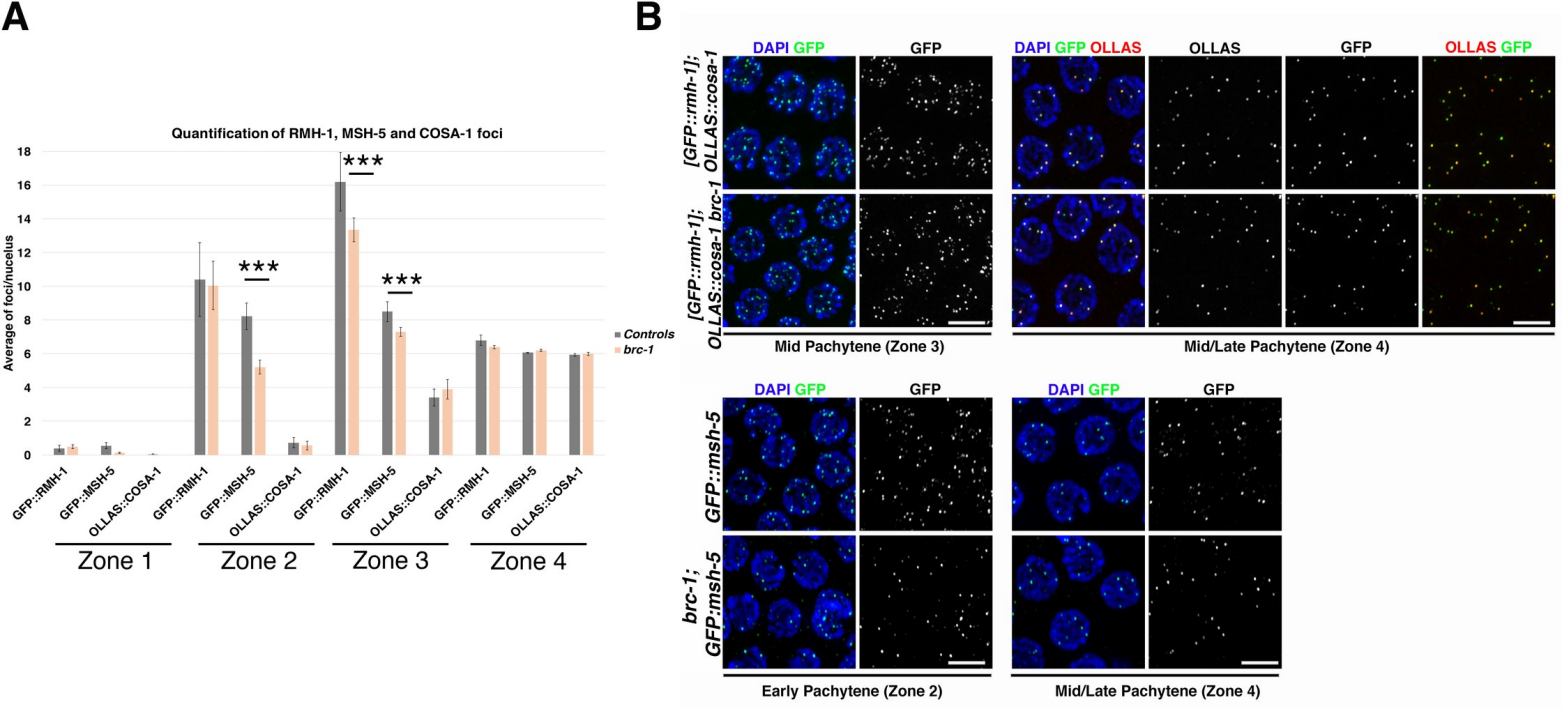
Analysis of recombination markers in *brc-1* mutants. (A) Quantification of GFP::RMH1, GFP::MSH-5 and OLLAS::COSA-1 markers in *brc-1 brd-1* mutants and control animals. Gonads were divided into four equal regions from the transition zone to the late pachytene stage. The average number of foci per nucleus from at least three gonads per genotype is shown. For GFP::RMH-1 and OLLAS::COSA-1 quantification, the number of nuclei scored for each gonad region in the controls (and *brc-1 brd-1* mutants) were: zone 1, 242 (334); zone 2, 185 (244); zone 3, 181 (214); zone 4, 124 (136). For GFP::MSH-5 quantification, the equivalent numbers were: zone 1, 230 (403); zone 2, 210 (410); zone 3, 165 (303); zone 4, 121 (147). Error bars show S.E.M. and *** indicate *p<0*,*0001* statistical significance as calculated by T test. (B) Representative nuclei at different meiotic stages co-stained for GFP::RMH-1 with OLLAS::COSA-1 (upper panels) or GFP::MSH-5 (lower panels). Scale bar, 5 μm. Note that both GFP::RMH-1 and GFP::MSH-5 are expressed in fewer foci in early and mid-pachytene but not in late pachytene in *brc-1 brd-1* mutants.

BRC-1 is dispensable for establishing synapsis and chiasmata; however, *brc-1* mutant germlines have a higher number of and more persistent RAD-51-labeled recombination intermediates compared with the wild type [46, 47]. Impaired BRC-1 localization, and probably also impaired function, in CO-defective mutants leads to the formation of abnormal chromosome structures in diakinesis nuclei, possibly due to deficient IS repair [47]. DSB repair during meiosis is channelled into both CO and NCO pathways. Since it has been suggested that BRC-1 might preferentially function in NCOs [47], we investigated whether other factors involved in resolving the recombination intermediates required for both CO and NCO repair might also be affected. In somatic cells, the BTR complex, formed by BLM, RMI1 and TOP3A, mediates efficient resolution of recombination intermediates by promoting the dissolution of double Holliday junctions to yield non-CO products [72–74]. The *C*. *elegans* RMI1orthologue, RMH-1, accumulates in many foci during meiotic prophase, possibly labelling all recombination intermediates. At late pachytene transition, the number of RMH-1 foci is reduced to roughly six per nucleus which co-localize with COSA-1, MSH-5 and ZHP-3. Lack of RMH-1 causes a drastic reduction in chiasmata formation due to impaired COSA-1 and MSH-5 loading. However, in CO-deficient backgrounds such as *cosa-1*, *msh-5* and *zhp-3* mutants, RMH-1 is still recruited in early pachytene but is not retained until late pachytene. Therefore, it has been postulated that RMH-1 functions in both the CO and NCO pathways [75]. We scored COSA-1, MSH-5 and RMH-1 nuclear localization in *brc-1* mutants in nuclei in the transition zone to late pachytene stage. Interestingly, GFP::MSH-5 accumulation was mildly, although significantly, reduced in early and mid-pachytene, with a similar, albeit less prominent, behaviour for GFP::RMH-1 (Fig 7A and 7B).

By late pachytene however, both proteins had been recruited into six foci, together with COSA-1, suggesting that BRC-1 might influence early processing of recombination intermediates, although formation of chiasmata was not affected.

### BRC-1-BRD-1 promote RAD-51 recruitment in absence of synapsis

**Fig 8:**
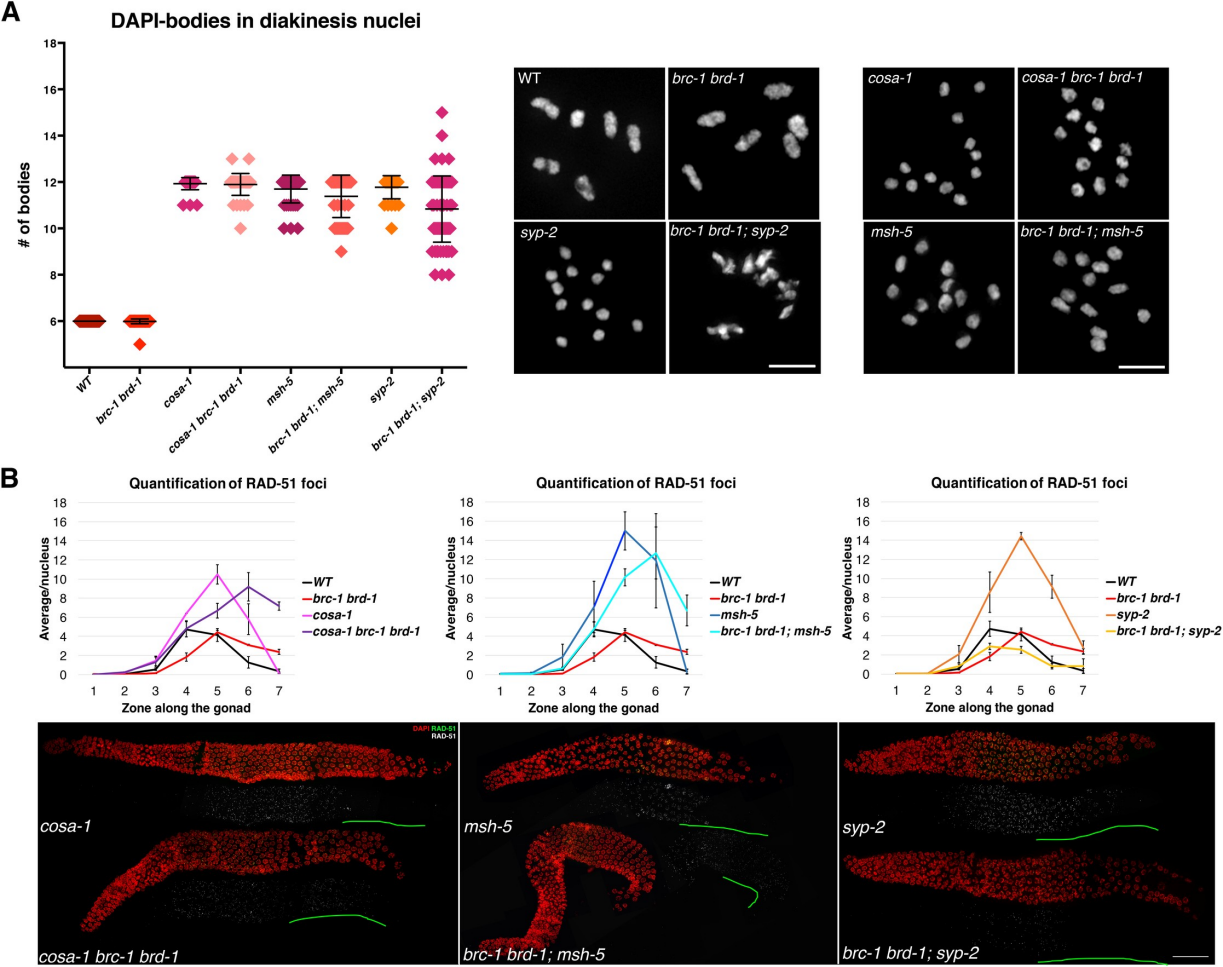
Loss of BRC-1 in CO-defective mutants differently influences RAD-51 loading in presence or absence of SC. (A) Left: quantification of DAPI-stained bodies in diakinesis nuclei in different genotypes. Number of diakinesis nuclei scored: WT, 44; *brc-1 brd-1*, 46; *cosa-1*, 42; *cosa-1 brc-1 brd-1*, 48; *msh-5*, 43; *brc-1 brd-1; msh-5*, 55; *syp-2*, 31; *brc-1 brd-1; syp-2*, 79. Right: representative images of DAPI-stained diakinesis nuclei in the genotypes quantified. Scale bar, 3 μm. (B) Top. Quantification of RAD-51 foci per nucleus throughout the germline. Each gonad was divided into seven equal zones and RAD-51 foci were counted in each nucleus. Data show the average of at least three gonads for each genotype. Number of nuclei scored from zone 1 to zone 7 in different genetic backgrounds: WT– 154, 226, 189, 157, 113, 95, 92; *brc-1 brd-1*–173, 189, 186, 156, 136, 125, 72; *cosa-1*–162, 225, 179, 165, 142, 107, 124; *cosa-1 brc-1 brd-1*–347, 335, 285, 274, 228, 175, 149; *msh-5*–205, 179, 120, 115, 94, 74, 66; *brc-1 brd-1; msh-5*–211, 237, 197, 194, 171, 114, 92; *syp-2*–244, 265, 241, 233, 190, 131, 118; *brc-1 brd-1; syp-2*–226, 305, 275, 297, 254, 161, 147. Error bars show S.E.M. Bottom. Representative examples of whole-mount gonads stained with DAPI and RAD-51, showing extended RAD-51 accumulation in both *cosa-1 brc-1 brd-1* and *brc-1 brd-1; msh-5* mutants, whereas dramatic reduction of RAD-51 is observed in *brc-1 brd-1; syp-2* mutants. Green lines indicate regions affected. Scale bars, 30 μm.

Given that BRC-1 and BRD-1 loading is regulated by synapsis and the establishment of COs, and that a lack of BRC-1 might affect the processing of NCOs rather than COs, we next assessed the effects of BRC-1 depletion in genetic backgrounds defective in chiasmata formation, which hence rely solely on NCOs to repair meiotic DSBs. We analyzed DAPI-stained bodies in diakinesis nuclei from *cosa-1 brc-1 brd-1*, *brc-1 brd-1; msh-5* and *brc-1 brd-1; syp-2* mutants and observed the presence of aberrant chromatin structures (Fig 8A), as previously reported [46, 47].

As abnormalities in diakinesis nuclei can result from impaired RAD-51-dependent repair of meiotic DSBs [24, 31, 76], we sought to analyze whether lack of function of the BCD complex altered RAD-51 dynamics. To this end, we quantified RAD-51 in the above-mentioned mutant backgrounds. Failure to convert recombination intermediates into mature CO products has been linked to increased RAD-51 levels and its delayed removal during meiotic prophase due to either excessive DSB induction or slower processing of recombination intermediates [5, 6, 15, 16], which are eventually channelled into alternative repair pathways (e.g. IS repair) [7]. In fact, *cosa-1*, *msh-5* and *syp-2* mutants all accumulated high levels of RAD-51, which disengaged from chromatin in mid- and late-pachytene (Fig 8B) [7, 20]. Remarkably, removal of BRC-1 from *cosa-1* and *msh-5* mutants had different effects on RAD-51 dynamics compared to *syp-2*: in all these strains, there were fewer RAD-51 foci in early pachytene (Fig 8B, zone 5) compared with *cosa-1*, *msh-5* and *syp-2* single mutants; however, in *cosa-1 brc-1 brd-1* and *brc-1 brd-1; msh-5* mutants RAD-51 accumulation was dramatically prolonged until diplotene entry whereas in the *brc-1 brd-1; syp-2* mutants RAD-51 staining was overall dramatically reduced (Fig 8B, S1–S3 Tables). Aberrant chromosome structures occurred at a particularly high frequency in *brc-1 brd-1; syp-2* mutants, consistent with the severe reduction in RAD-51 loading in pachytene nuclei (Fig 8A).

To be efficiently loaded to the single-stranded DNA (ssDNA) tails generated after resection, RAD-51 must be exchanged with RPA (RPA-1 in worms), which coats ssDNA tails to stabilize them and prevent DNA from self-winding [77, 78]. Given the altered dynamics of RAD-51 expression, we decided to analyze RPA-1 [79] to assess whether ssDNA was properly formed and processed. RPA-1 highly accumulated in *brc-1 brd-1; syp-2RNAi*, forming bright, discrete foci in both early and late pachytene cells (S6A Fig). This indicates that blocking BRC-1 function in synapsis-deficient mutants prevents efficient RAD-51 loading at meiotic DSBs, causing accumulation of unrepaired ssDNA as evidenced by RPA-1 foci formation. The *cosa-1 brc-1 brd-1* mutants did not show RPA-1 accumulation in early pachytene and late pachytene cells displayed occasional, weak foci, suggesting perhaps only a mild delay in the processing of recombination intermediates which eventually takes place faithfully, as shown by the formation of largely normal DAPI-bodies in diakinesis nuclei in the *brc-1 brd-1; msh-5* mutants (Figs S6B and 8A). Thus, in CO-defective mutants, BCD-dependent regulation of RAD-51 dynamics is altered by the presence of the SC.

### Efficient RAD-51-mediated repair upon exogenous DSB induction requires functional BRC-1-BRD-1

**Fig 9:**
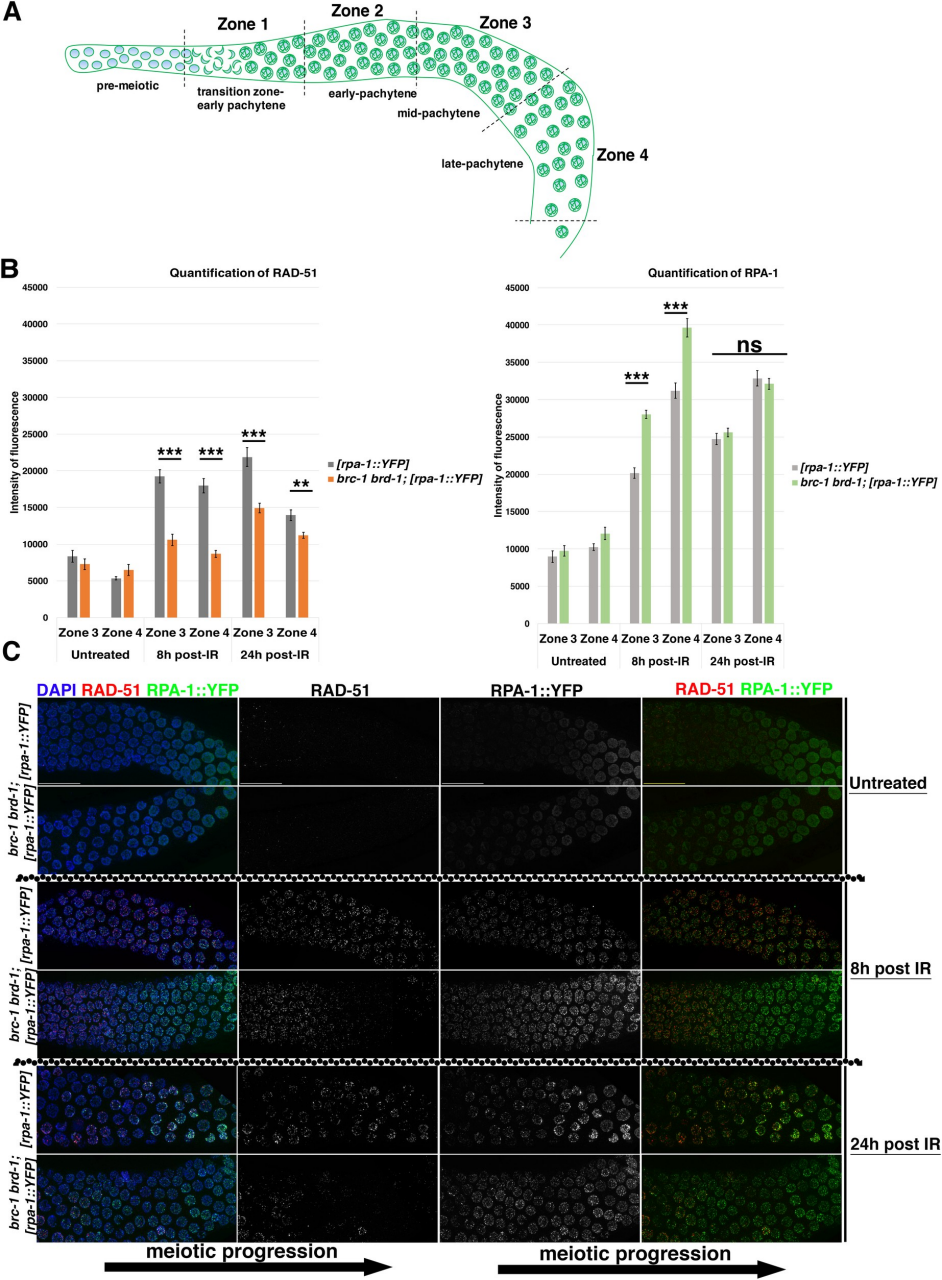
Efficient accumulation/exchange of RAD-51 and RPA-1 upon exogenous DNA damage requires BRC-1–BRD-1 function. (A) Schematic representation of the germline, divided into four equal regions, starting from transition zone and ending at diplotene entry, in which the quantification of RAD-51 and RPA-1 accumulation was performed. (B) Time course analysis of RAD-51 and RPA-1::YFP accumulation in irradiated *brc-1 brd-1* and controls. Worms were irradiated with 75 Gy IR and analyzed after 8 and 24 hours. The charts show quantification in zone 3 and 4, in which defective loading/retention of RAD-51 was observed. Germlines were acquired with the same settings, and quantification of fluorescence intensity was performed in Fiji. T test was conducted to assess statistical significance between the samples analyzed: *** p<0,0001, ** p = 0,001, ns = non-significant). (C) Representative examples of mid-pachytene regions from controls and *brc-1 brd-1* mutants analyzed at different times post-irradiation. Meiotic progression from early towards late pachytene is indicated by black arrow. Scale bars, 30 μm.

Exposure of *brc-1* and *brd-1* mutants to IR causes dose-dependent hypersensitivity which eventually culminates in full sterility, possibly due to the formation of highly unstructured chromatin bodies in diakinesis nuclei [46]. These structures resemble those formed upon BRC-2/BRCA2 depletion, which in worms is essential for RAD-51 loading [31, 76], and COM-1/Sae2 depletion, which promotes DSB resection [68, 80]. Both mutants lack RAD-51 recruitment onto DNA during meiotic prophase I. We therefore sought to investigate whether the aberrant chromatin masses observed in irradiated *brc-1 brd-1* mutants were caused by impaired RAD-51 recruitment. We analyzed RAD-51 and RPA-1 loading at two different time points post-irradiation. At 8h post-irradiation, we observed a dramatic reduction in RAD-51 focus formation specifically in mid- and late-pachytene nuclei of *brc-1 brd-1* mutants, along with enhanced RPA-1 levels (Fig 9B and 9C).

At 24 hours post-irradiation, RAD-51 was still markedly reduced (especially in mid-pachytene stage) while RPA-1 levels in *brc-1 brd-1; [rpa-1*::*YFP]* mutants were comparable to the controls (Fig 9B and 9C). Prompted by these results, we decided to analyze the loading dynamics of BRC-1::HA and RAD-51 after IR exposure to assess whether exogenous DSB formation affected the mutual spatio-temporal regulation of these proteins. Under homeostatic conditions, BRC-1 and RAD-51 localization did not overlap prior to BRC-1 enrichment in the SC, which occurs after RAD-51 disappearance (S7A and S7B Fig). At 1 hour post-irradiation, BRC-1::HA started to form discrete chromatin-associated foci in pre-meiotic nuclei, often in close proximity to (but not co-localizing with) RAD-51 foci (S7A and S7B Fig). Although abundant RAD-51 accumulation was triggered by IR exposure throughout the germline, BRC-1::HA levels were only modestly increased. However, western blot analysis revealed a shift in BRC-1::HA migration after IR which remained unchanged throughout the time course (S7C Fig), suggesting that exogenous DNA damage might elicit post-translational modifications of BRC-1. Western blot analysis also showed a slight increase in BRC-1::HA abundance, confirming our immunofluorescence data (S7A Fig). Samples analyzed 8 hours after IR revealed robust BRC-1 and RAD-51 co-localization in nuclei residing in the mitotic tip; however, as at the earlier time point, no clear co-localization was observed in pachytene nuclei (S7B Fig). At 24 hours post-irradiation, BRC-1::HA foci in the mitotic nuclei had largely disappeared and bright RAD-51 foci were observed only in enlarged, G2-arrested nuclei that were still undergoing repair; in contrast, bright RAD-51 foci co-localizing with BRC-1 were occasionally seen in non-arrested nuclei. Taken together, our observations revealed that BRC-1 accumulation in the germline is modulated by exogenous DNA damage and that the clear BRC-1 and RAD-51 co-localization observed only in mitotic nuclei was cell cycle dependent.

## Discussion

Our study sheds new light on the expression dynamics of the *C*. *elegans* BRC-1–BRD-1 heterodimer during meiotic prophase I and reveals that the BCD complex regulates RAD-51 accumulation in the germline under both homeostatic conditions of growth and upon genotoxic stress. We show that in contrast to mammalian meiosis, where BRCA1 is loaded exclusively at asynapsed chromosome regions during spermatogenesis and oogenesis in pachytene cells [48, 49, 54], in worms both BRC-1 and BRD-1 are expressed throughout meiotic prophase I and are progressively enriched to the short arms of bivalents, in a CO-, SC- and PLK-2-dependent manner. Our data provide the first evidence that BRC-1 and BRD-1 form complexes *in vivo* with the pro-CO factor MSH-5 and the SC central element SYP-3. Taken together, our findings provide new insight into the meiotic functions of BRC-1 and BRD-1 and show that the BCD complex is essential for preserving genome integrity and stimulating HR during gametogenesis.

### The BCD complex functions at the interface of synapsis and recombination

BRC-1 and BRD-1 display a highly dynamic localization pattern during meiotic prophase I progression, shifting from a rather diffuse accumulation at early stages to a robust association with the SC in late pachytene, which culminates in retention of the BCD complex at the short arm of the bivalent (Figs 1–3). Remarkably, accumulation of BRC-1–BRD-1 at specific chromosomal subdomains occurs prior to retraction of the SC central elements to those domains but is concomitant with recombination factor-dependent enrichment of PLK-2 at the SC (Fig 2B) [62, 64], suggesting that the BCD complex is actively targeted to the region surrounding the CO rather than passively recruited following SC remodelling.

Importantly, recruitment of BRC-1–BRD-1 to the short arm of the bivalent depends on PLK-2 function, as chromatin association of BRC-1 is strongly impaired in *plk-2* null mutants: enrichment of BRC-1::HA at the SC is extremely delayed and occurs only in a short region before diplotene entry in *plk-2* mutants (Fig 2C). Furthermore, rather than displaying a gradual retraction towards the short arm of the bivalent, BRC-1::HA abruptly formed discrete foci (presumably at the CO sites which are still formed in absence of PLK-2) and was largely retained in the nucleoplasm (Fig 2C). We envision a scenario where the localization of BRC-1 at the presumptive CO sites in *plk-2* null mutants is accomplished *via* the physical interaction with the CO machinery (Fig 4). It has been shown that albeit reduced, COs still form in absence of PLK-2, which are dependent on PLK-1 activity [60, 61]. Therefore, we hypothesize that residual BRC-1 accumulation observed in *plk-2* mutants might be dependent on PLK-1.

Our data favour a model in which the SC also exerts an essential role for the recruitment of the BCD complex onto the chromosomes and its later accumulation at the CO site, due to the local concentration of recombination factors. In fact, BRC-1 recruitment to the SC is not prevented in *msh-5* or *spo-11* mutants (both of which are defective in CO formation but proficient in synapsis establishment). However, similar to ZHP-3, BRC-1 fails to retract (Fig 5A and 5C). Irradiation of *spo-11* mutants restored BRC-1 and ZHP-3 redistribution to the short arms of bivalents (Fig 5B), confirming that CO establishment *per se* is the key trigger of local BCD complex enrichment. Abrogation of synapsis dramatically changed the BRC-1 expression pattern: it remained punctate throughout meiotic prophase I and displayed extensive co-localization with RAD-51 specifically in late pachytene cells (Fig 6A and 6B). However, in mutants in which only one chromosome pair was asynapsed, such as *him-8* and *zim-2* mutants, BRC-1 was not loaded onto the unsynapsed chromosomes but localization was normal on the remaining ones (Fig 6C and 6D).

Our data suggest that the SC alone is not sufficient for proper BRC-1 and BRD-1 loading but it rather cooperates with PLK-2 to regulate the function and localization dynamics of the BCD complex. In fact, *plk-2* mutants still display extensive regions of synapsis throughout the germline, however recruitment of BRC-1 is aberrant. If successful loading of the BCD complex was solely dependent on the SC loading, then we would have expected to find unperturbed BRC-1 localization at the synapsed regions, which instead was not the case. This suggests that localization of BRC-1 and BRD-1 undergoes a complex and tightly controlled regulation. It was recently shown that PLK-2 plays a pivotal role in modulating the physical state of the SC in response to recombination and that absence of synapsis impairs PLK-2 redistribution from the nuclear envelope to chromosome subdomains [62–64], which might explain the different BRC-1 localization patterns in *syp-2* mutants. Different BRD-1 and BRC-1 localization patterns were as well observed in *htp-1* and *prom-1* mutants, both characterized by extensive non-homologous synapsis, in which the BCD complex accumulated in bright agglomerates in the nucleus (S5A and S5B Fig). It is important to mention here, that *htp-1* null mutants display an extremely reduced PLK-2 accumulation at the nuclear envelope at meiosis onset and absence of SC-associated PLK-2 in late pachytene nuclei [53], suggesting, once again, that SYP loading *per se* is not sufficient to recruit BRC-1–BRD-1 onto the SC and that PLK-2 might exert further roles in regulating BRC-1 (and by assumption, BRD-1) localization possibly through phosphorylation-dependent modifications. However, compared to *plk-2* null mutants in which a residual accumulation of BRC-1 at the SC still occurs, in *htp-1* mutants (which also lack PLK-2 loading in late pachytene nuclei) both BRC-1::HA and BRD-1 entirely fail to be recruited along the chromosomes, indicating that the requirements for proper localization of the BCD complex dwell in multiple layers of control and that ectopic polymerization of the SC between non-homologous chromosomes dramatically perturbs BRC-1 and BRD-1 association with the chromatin.

Moreover, despite their clear enrichment at the putative CO sites, our data, as well as previous studies [46, 47], show that chiasmata formation occurs normally in absence of a functional BCD complex, indicating that recruitment of BRC-1-BRD-1 at CO-designation sites is not essential for eliciting COs but it might rather occur in response to their establishment. Intriguingly though, Li and colleagues show in the accompanying study that the recombination landscape is altered in *brc-1* mutants, as recombination rate increases in the center of chromosomes at the expenses of the terminal regions, which instead bear the majority of recombination events in WT worms. Further, under compromised meiosis (i.e. *zim-1* mutants), BRC-1 can promote formation of extra-COs in the presence of chromosome pairs lacking a chiasma. This would indicate that BRC-1 and BRD-1 exert a regulatory activity on the recombination intermediates and can act as a switch in the choice of CO versus NCO pathway.

### Crosstalk between the BCD complex and RAD-51 is governed by the SC

Blocking BRC-1 function had opposing effects on the progression of recombination intermediates in *cosa-1* and *msh-5*, compared to *syp-2* (CO-defective) mutants. RAD-51 accumulation was exacerbated in *cosa-1* and *msh-5* single mutants and largely suppressed in *syp-2* mutants (Fig 8), leading to the formation of aberrant chromatin masses in diakinesis nuclei as previously reported [47]. Based on genetic data, BRC-1 function was previously postulated to be essential for IS repair of meiotic DSBs [47, 81, 82]; our data corroborate this model. In *cosa-1 brc-1 brd-1* and *brc-1 brd-1; msh-5* mutants, the presence of an intact SC might still impose a homologue-biased constraint for an inter-homologue, CO-independent pathway that relies on RAD-51-mediated repair but not on BRC-1 function. However, in the absence of synapsis, repair of recombination intermediates is probably channelled entirely through the IS repair pathway given that the sister chromatid is the only available repair template: in line with this, our data show that in fact SC depletion triggers association of BRC-1 with RAD-51 in late pachytene cells at presumptive repair sites, thereby likely promoting HR-mediated repair. We also observed fewer RAD-51 foci in *brc-1 brd-1; syp-2* mutants during early pachytene, suggesting that BRC-1 is nonetheless required to (directly or indirectly) promote efficient RAD-51 loading at meiosis onset, although co-localization with RAD-51 in early stages might be very transient. We observed that lack of BRC-1 mildly, although significantly, impacts on the loading of recombination markers such as MSH-5 and RMH-1 in early pachytene, suggesting that even in the presence of the SC, BRC-1–BRD-1 function might be required to efficiently promote the processing of recombination intermediates. Moreover, in *brc-1 brd-1* mutants exposed to exogenous DSB induction, RAD-51 is not efficiently retained in mid- and late-pachytene cells (Fig 9). This is not due to impaired resection, as shown by the abundant recruitment of RPA-1, which stabilizes ssDNA. However, RAD-51 loading is comparable to controls in later stages, suggesting that stabilization, rather than loading *per se*, might require the action of the BCD complex. This is in line with the findings reported by Li et al. (see accompanying manuscript).

When we scored BRC-1 levels after exposure to IR, we detected a slight increase in abundance but a marked difference in protein migration on western blots (S7A and S7C Fig), suggesting that exogenous DNA damage may promote post-translational modification of BRC-1. Importantly, despite dramatically enhanced RAD-51 levels upon irradiation, we observed clear co-localization with BRC-1 only in mitotic cells and not during pachytene, once again confirming that these proteins co-localize only when the SC is indeed absent (S7B Fig).

Our findings suggest that the BCD complex responds to both synapsis and recombination and that the SC might act as a docking site for the BRC-1–BRD-1 complex to modulate its function in promoting meiotic DNA repair.

## Materials and methods

**Worm strains** All worm strains used in this study were grown at 20°C and the N2 Bristol strain was used as the wild type. The following mutant alleles and tagged lines were used: LGI: *syp-3(ok758)/hT2[bli-4(e937) let-*?*(q782) qIs48]*, *prom-1(ok1140)*, *rmh-1(jf54)*, *plk-2(ok1936)*; LGII: *[GFP*::*rmh-1]* [75], *[GFP*::*syp-3]* [67], *[GFP*::*cosa-1]* [20], *oxTi933 [eft-3p*::*GFP*::*2xNLS*::*tbb-2 3'UTR + Cbr-unc-119(+)]*; LGIII: *brc-1(tm1145)*, *brc-1(KO)* (this study), *brc-1*::*HA* (this study), *GFP*::*brc-1* (this study), *brd-1(gk297)*, *brd-1*::*HA* (this study), *brd-1(dw1)*, *cosa-1(tm3298)/qC1[dpy-19(e1259) glp-1(q339) qIs26] III*, *OLLAS*::*cosa-1* (this study), *com-1(t1626)/hT2[bli-4(e937) let-*?*(q782) qIs48];* LGIV: *spo-11(ok79)/nT1[unc-*?*(n754) let-*?*]*, *him-8(tm611)*, *zim-2(tm574)*, *msh-5(me23)/nT1[unc-*?*(n754) let-*?*]*, *GFP*::*msh-5* (this study), *msh-5*::*2xHA* (this study), *htp-1(gk174)/nT1[unc-*?*(n754) let-*?*]*, *dpy-13(e184) rad-51(lg8701)/nT1 [let-*?*(m435)]*; LGV: *syp-2(ok307)/nT1[unc-*?*(n754) let-*?*(m435)]*. No information is available on the chromosomal integration of *[rpa-1*::*YFP]* [79].

The DW102 strain carrying the *brc-1(tm1145)* deletion allele also contains the closely linked *brd-1(dw1)* deletion allele, which was not previously reported. We made sure that all of the strains generated during this study were carrying the two mutations linked in order to assess the phenotypes caused by the impaired function of the BCD complex.

**Viability assessment** Worms were individually picked and moved onto new plates every 12h for three days. Dead eggs and viable larvae were scored 24h after the mother had been moved, whereas male progeny was counted three days later. Embryos viability was calculated as the number of hatched over the total number of laid eggs and percentage of males was calculated as the total number of male progeny over hatched eggs.

### RNA interference

RNAi for *syp-2* was performed employing the clone available in the Ahringer library. A single colony from a freshly struck glycerol stock on plates containing 100 mg/ml ampicillin and 12.5 mg/ml of tetracycline was inoculated in 20 ml of LB containing 100 mg/ml of ampicillin and grown overnight at 37°C. The following day, the bacteria were concentrated in 2 ml of LB containing 100 mg/ml ampicillin and 100μl of culture were seeded per plate, containing 100 mg/ml of ampicillin and 1mM IPTG. The same procedure was followed for the bacteria containing the pL4440 empty vector as a control. Plates were left at 37°C overnight to induce dsRNA and the following day, L4 *[rpa-1*::*YFP]* and *brc-1; [rpa-1*::*YFP]* worms were placed on the induced plates. F1 worms at L1 stage were picked and transferred onto freshly induced plates three days later. Worms were dissected 24h post L4 stage. The RNAi was performed at 20°C and only the germlines displaying 12 univalents in diakinesis nuclei, indicative of successful *syp-2* depletion, were analyzed for YFP staining.

### Cytological procedures

For cytological analysis of whole-mount gonads, age-matched worms (20–24 hours post-L4 stage) were dissected in 1× PBS on a Superfrost Plus charged slide and fixed with an equal volume of 2% PFA in 1× PBS for 5 min at room temperature. Slides were freeze-cracked in liquid nitrogen and then incubated in methanol -20°C for 5 min, followed by three washes in PBST (1× PBS, 0.1% Tween) at room temperature. Slides were blocked for 1 hour at room temperature in PBST containing 1% BSA and then primary antibodies were added in PBST and incubated overnight at 4°C. Slides were then washed in PBST at room temperature and secondary antibodies were applied for 2 hours. After three washes 10 min each in PBST, 60μl of a 2 μg/ml stock solution of DAPI in water was added to each slide and stained for 1 min at room temperature. Samples were washed again for at least 20 min in PBST and then mounted with Vectashield. For detection of GFP::MSH-5, worms were dissected and fixed in 1× EGG buffer containing 0.1% Tween instead of PBST. Detection of [RPA-1::YFP] was performed as previously described [83]. Primary antibodies used in this study were: mouse monoclonal anti-HA tag (pre-absorbed on N2 worms to reduce non-specific binding; 1:100 dilution; Covance), rabbit anti-HA tag (1:250 dilution; Invitrogen), rabbit anti-BRD-1 (pre-absorbed on *brd-1(dw1)* worms to reduce non-specific binding; 1:500 dilution) [52], chicken anti-SYP-1 (1:500 dilution) [53], guinea pig anti-HTP-3 (1:500 dilution) [59], mouse monoclonal anti-GFP (1:500 dilution; Roche), guinea pig anti-ZHP-3 (1:500 dilution) [21], rabbit anti-OLLAS tag (pre-absorbed on N2 worms to reduce non-specific binding; 1:150 dilution; GenScript), rabbit anti-RAD-51 (1:10,000 dilution; SDIX) and rabbit anti-PLK-2 (1:500 dilution) [84]. Appropriate secondary antibodies were conjugated with Alexa Fluor 488 or 594 (1:500 dilution) or with Alexa Fluor 647 (1:250 dilution). Images were collected as z-stacks (0.3 μm intervals) using an UPlanSApo 100x NA 1.40 objective on a DeltaVision System equipped with a CoolSNAP HQ2 camera. Files were deconvolved with SoftWORx software and processed in Adobe Photoshop, where some false colouring was applied. Samples acquired by super-resolution microscopy (Fig 3D and 3E) were prepared as previously reported [62] without modifications and imaged with a DeltaVision OMX. For quantification of RPA-1::YFP and RAD-51 in Fig 9, samples were acquired with same settings and identically adjusted in Fiji. Gonads were divided into four equal regions starting from transition zone and ending before diplotene entry. A circle with a fixed area was drawn in Fiji and intensity of fluorescence was scored in each nucleus for all regions.

### Biochemistry

For whole-cell protein extraction, 200 age-matched animals (24 hours post-L4 stage) were picked into 1× Tris-EDTA buffer (10 mM Tris pH 8, 1 mM EDTA) containing 1× protein inhibitor cocktail (Roche) and snap-frozen in liquid nitrogen. After thawing, an equal volume of 2× Laemmli buffer was added. Samples were boiled for 10 min, clarified and separated on pre-cast 4–20% gradient acrylamide gels (Bio Rad).

Fractionated protein extracts for western blotting and immunoprecipitation were prepared as previously reported [53]. Western blotting used 50 μg of protein samples from each fraction, whereas for immunoprecipitation assays at least 1 mg of extract from pooled soluble nuclear and chromatin-bound fractions was used. For the inputs, 5% of the amounts used for IPs was run. Immunoprecipitation of GFP-tagged proteins was performed with agarose GFP-traps (Chromotek). For all immunoprecipitation experiments, pre-equilibrated beads in buffer D (20% glycerol, 0.2 mM EDTA pH 8, 150 mM KCl, 20 mM Hepes-KOH pH 7.9, 0.2% Triton X-100, supplemented with protease inhibitor cocktail from Roche), were incubated with the extracts over night at 4°C in mild agitation. The following day, beads were separated from immuno-depleted extracts, washed extensively in buffer D, re-suspended in 40 μl of 2x Laemmli Buffer (Sigma) and boiled for 10 minutes to recover immunocomplexes. Beads were pelleted by centrifugation at maximum speed for 1 min and surnatants were run in 1× SDS-Tris-glycine buffer on a pre-cast 4%-20% TGX gels (BioRad). Proteins were transferred onto nitrocellulose membrane for 1 hour at 4°C at 100V in 1× Tris-glycine buffer containing 20% methanol. Membranes were blocked for 1 hour in 1× TBS containing 0.1% Tween (TBST) and 5% milk; primary antibodies were added into the same buffer and incubated overnight at 4°C. Membranes were then washed in 1× TBST and then incubated with appropriate secondary antibodies in TBST containing 5% milk for 1 hour at room temperature. After washing, membranes were incubated with ECL (Amersham) and developed with a ChemiDoc system (BioRad). To detect phosphorylated CHK-1^S345^, TBST containing 5% BSA instead of milk was used for both blocking and antibody dilution. The following antibodies were used for western blotting: mouse monoclonal anti-HA tag (1:1000 dilution; Cell Signalling), rabbit anti-HA tag (1:500 dilution; Invitrogen), anti-BRD-1 [52] (1:1000 dilution), chicken anti-GFP (1:4000 dilution; Abcam), mouse anti-GAPDH (1:10,000 dilution; Ambion), goat anti-Actin (1:3000; Santa Cruz), rabbit anti-Histone H3 (1:100,000 dilution; Abcam); rabbit anti-phospho-CHK-1^S345^ (1:1000 dilution; Cell Signalling), HRP-conjugated anti-mouse (1:2500 dilution) and anti-rabbit (1:25,000 dilution; both Jackson ImmunoResearch), HRP-conjugated anti-chicken and anti-goat (both 1:10,000 dilution; Santa Cruz).

### Irradiation

Age-matched worms (24 hours post-L4 stage) were exposed to the indicated dose of IR with a Gammacell irradiator containing a ^137^Cs source. For viability screening, irradiated worms were allowed to lay eggs for 24 hours and then removed; hatched versus unhatched eggs were scored the following day. For cytological analysis, worms were dissected and immunostained at the indicated times.

**CRISPR-Cas9 tagging** A C-terminal HA-tag was inserted at the endogenous locus encoding the *brc-1* gene by using a CRISPR-Cas9 based approach as in [85]. Briefly, a 2,335 base pairs region (8541 to 10875 from the ATG) of the *brc-1* locus was amplified by PCR from genomic DNA and cloned in pCR2.1 vector (TA cloning, Invitrogen). The plasmid obtained was used as a template to insert a 27 base pairs DNA fragment encoding the HA-tag before the STOP codon with the Gibson mutagenesis kit (NEB). The full insert, now including the HA-tag, was amplified by PCR and cloned in pCFJ104 at the Bgl II site. N2 worms were injected with a mix containing 25 ng/μl of pCFJ90 (*Pmyo-2*::*mCherry*; Addgene) which was used as a co-injection marker, 200 ng/μl of the sgRNA vector (pUC57, in which the *unc-119* sgRNA sequence was replaced with 5´-AAATGGAAAATTAATCCTGC-3´sequence), 175 ng/μl of the *Peft-3*Cas9-SV40 NLS*tbb-2*3´-UTR) and 220 ng/μl of the donor vector. mCherry positive worms were individually picked and genotyped to identify insertion events. To generate the *GFP*::*msh-5* and the *GFP*::*brc-1* strains, the GFP was amplified with a pair of primers carrying 25 bases of homology to each side of the region flanking the ATG of the *msh-5* or the *brc-1* genes. The sequence 5´-TGGTTCAAATGTCCACTCGA-3´ was used as a crRNA (Dharmacon) for *msh-5* and the 5´-AGATGGCAGATGTTGCACTG -3´ for *brc-1*. The tagging strategy was the same used in [86]. The same experimental design was followed to tag the endogenous *cosa-1* locus with a 5´- OLLAS-tag (tag sequence: SGFANELGPRLMGK). A 200 base pairs DNA ultramer (IDT) carrying the OLLAS-tag immediately after the ATG was employed. The sequence 5´-AAGTGTCAATGTCAAGTTCT-3´ was used as a crRNA (Dharmacon). Synthetic 200 base pairs DNA ultramers (IDT) were employed to generate the *msh-5*::*2xHA* and the *brd-1*::*HA* as well: the crRNAs used were 5´-CGAACGATCTATCGTCTCAT-3´ and 5´-ACGGAAAATGGTTAATGTGG-3´ respectively. To generate a full deletion of the *brc-1* locus (*brc-1(KO)*), two sgRNAs were designed to target the beginning (5´-AGATGGCAGATGTTGCACTG-3´) and the end (5´-CGATTCGATAGGCTGCCTGC-3´) of the *brc-1* gene. A repair template carrying the 5´-UTR directly in fusion with the STOP codon was synthesised (IDT). The 9.713 base pairs deletion allele obtained was sequenced to assess deletion boundaries. The resulting sequence of the *brc-1* locus in the *KO* allele is 5´-…atgaaatgttatttgtttaaaatttaattt**CAG**aggat**TAA**ttttccatttcttcttcttctttctttgttc…-3´, where “CAG” are the bases immediately preceding the ATG, and the “TAA” is the STOP-codon. All the strains generated by CRISPR were sequenced to ensure fidelity of the insertion and backcrossed to N2 worms at least twice prior usage.

## Supporting information

S1 Fig BRD-1 and BRC-1 localization is mutually dependent. (A) Anti-BRD-1 and anti-SYP-1 immunostaining in wild-type animals shows that the BRD-1 expression pattern is identical to the one observed for BRC-1::HA. Note enrichment on the SC and retraction to the short arms of bivalents. Scale bar, 30 μm. (B) Late pachytene nuclei stained with anti BRD-1 and SYP-1 antibodies reveal lack of BRD-1 in *brc-1(tm1145)* mutants. Scale bar, 5 μm. (C) Left: western blot analysis on whole worm extracts shows that BRD-1 is not expressed in *brc-1(tm1145)* and that both *brd-1(dw1)* and *brd-1(gk297)* are null alleles of *brd-1*. Asterisk indicates a non-specific doublet recognised by the anti BRD-1 antibody. Right: genotyping for *brd-1(dw1)* reveals *dw1* deletion in *brc-1(tm1145)* mutants and all the strains employed in this study. Numbers indicate different strains: 1-WT; 2-*brc-1(tm1145)*, 3-*brd-1(dw1)*, 4-*brc-1(tm1145); msh-5/nT1*, 5-*brc-1(tm1145); [rpa-1*::*YFP]*, 6-*brc-1(tm1145); GFP*::*msh-5*, 7-*brc-1(tm1145) OLLAS*::*cosa-1; GFP*::*rmh-1*, 8-*brc-1(tm1145); syp-2/nT1*, 9-*cosa-1(tm3298) brc-1(tmm145)/qC1*. (D) Top: late pachytene nuclei stained with HA antibodies, showing that BRC-1::HA is not detected in *brd-1(gk297)*. Bottom: BRD-1::HA is normally loaded in *brc-1(tm1145)* mutants. (E) Endogenous BRD-1 and BRD-1::HA are not loaded in *brc-1(KO)* knock outs, proving loading interdependency between BRC-1 and BRD-1. (F) Western blot analysis shows expression of BRD-1::HA and BRC-1::HA in the relevant genetic backgrounds. WT (N2) worms were used as negative controls and actin was used as loading control. Note that BRD-1::HA and BRC-1::HA displayed reduced levels in null *brc-1(KO)* and *brd-1(gk297)* mutants.
(TIF)

S2 Fig BRD-1 is enriched at the short arm of the bivalent. Late pachytene nuclei of *[GFP*::*cosa-1]* animals were stained for BRD-1, GFP and SYP-1. As previously observed for BRC-1::HA, BRD-1 is progressively enriched at the short arm of the bivalent, also containing COSA-1-labeled CO site. Scale bar, 5 μm.
(TIF)

S3 Fig Occasional spontaneous DSBs trigger recruitment of BRC-1::HA and ZHP-3 at the short arm of the bivalent in unirradiated *spo-11* mutants. Two examples of late pachytene-diplotene nuclei in non-irradiated *spo-11* mutants stained for BRC-1::HA and ZHP-3 showing retraction to the short arm of the bivalent. Scale bar, 5 μm.
(TIF)

S4 Fig Association of BRD-1 with the SC is largely disrupted in DSBs resection-defective *com-1* mutants. Mid-/late pachytene nuclei of the wild type (WT) and *com-1* mutant were stained for BRD-1. BRD-1 loading onto the SC is drastically reduced when DNA resection is impaired. Scale bar, 5 μm.
(TIF)

S5 Fig Non-homologous synapsis largely impairs loading of BRD-1 and BRC-1::HA leading to their nucleoplasmic accumulation. (A) Late pachytene nuclei in the wild-type (WT), *htp-1* and *prom-1* mutants were stained with BRD-1, SYP-1 and HTP-3. In both mutants, BRD-1 is largely excluded from the SC and forms nucleoplasmic agglomerates. (B) A similar staining pattern was observed for BRC-1::HA in *htp-1* null mutants. Scale bar, 5 μm.
(TIF)

S6 Fig RPA-1 accumulates in *brc-1 brd-1; syp-2*^*RNAi*^ but not in *cosa-1 brc-1 brd-1* mutants. (A) Impairment of *brc-1 brd-1* function upon synapsis deficiency causes accumulation of RPA-1::YFP in pachytene nuclei. EP = early pachynema, LP = late pachynema. (B) In *cosa-1 brc-1 brd-1* mutants, dim RPA-1::YFP foci were only occasionally detected in few cells in late pachynema, suggesting that in absence of COs, impaired function of BCD complex in presence of functional SC does not prevent RPA-1/RAD-51 exchange. This is consistent with defective RAD-51 loading observed in *brc-1 brd-1; syp-2* mutants but not in *cosa-1 brc-1 brd-1*. Scale bar, 5 μm.
(TIF)

S7 Fig Exogenous DNA damage increases BRC-1 levels and triggers its association with RAD-51 in mitotic nuclei. (A) Whole-mount gonads of irradiated and non-irradiated *brc-1*::*HA* worms immunostained for HA and RAD-51. Animals were exposed 75 Gy IR and analyzed at the indicated time points. (B) Representative nuclei from the pre-meiotic region (MT) and late pachytene (LP) stage of gonads analyzed at different times after IR. Note BRC-1::HA focus formation in pre-meiotic nuclei, along with robust co-localization with RAD-51 at 8 hours and occasionally at 24 hours post-irradiation. Scale bars, 5 μm. (C) Western blot analysis of whole-cell extracts shows a shift in BRC-1::HA migration after irradiation. Wild-type (WT) worms were used as negative control. Actin was the loading control and induction of phosphorylated CHK-1^Ser345^ was used as a positive control for irradiation. The ratio of BRC-1::HA to actin (HA/Actin) is shown as an abundance index.
(TIF)

S1 Table Statistical analysis of RAD-51 foci counts in *cosa-1 brc-1 brd-1* mutants and relative controls. T test was performed on RAD-51 foci number in different genotypes from transition zone to pachynema, corresponding to zone 4, 5, 6 and 7.
(DOCX)

S2 Table Statistical analysis of RAD-51 foci counts in *brc-1 brd-1; msh-5* mutants and relative controls. T test was performed on RAD-51 foci number in different genotypes from transition zone to pachynema, corresponding to zone 4, 5, 6 and 7.
(DOCX)

S3 Table Statistical analysis of RAD-51 foci counts in *brc-1 brd-1; syp-2* mutants and relative controls. T test was performed on RAD-51 foci number in different genotypes from transition zone to pachynema, corresponding to zone 4, 5, 6 and 7.
(DOCX)
