# Supplementary figures and images for: BRCA1-BARD1 associate with the synaptonemal complex and pro-crossover factors and influence RAD-51 dynamics during Caenorhabditis elegans meiosis
Source: PLoS Genet. 2018 Nov 1;14(11):e1007653. doi: 10.1371/journal.pgen.1007653 (PMC6211622; doi:10.1371/journal.pgen.1007653)

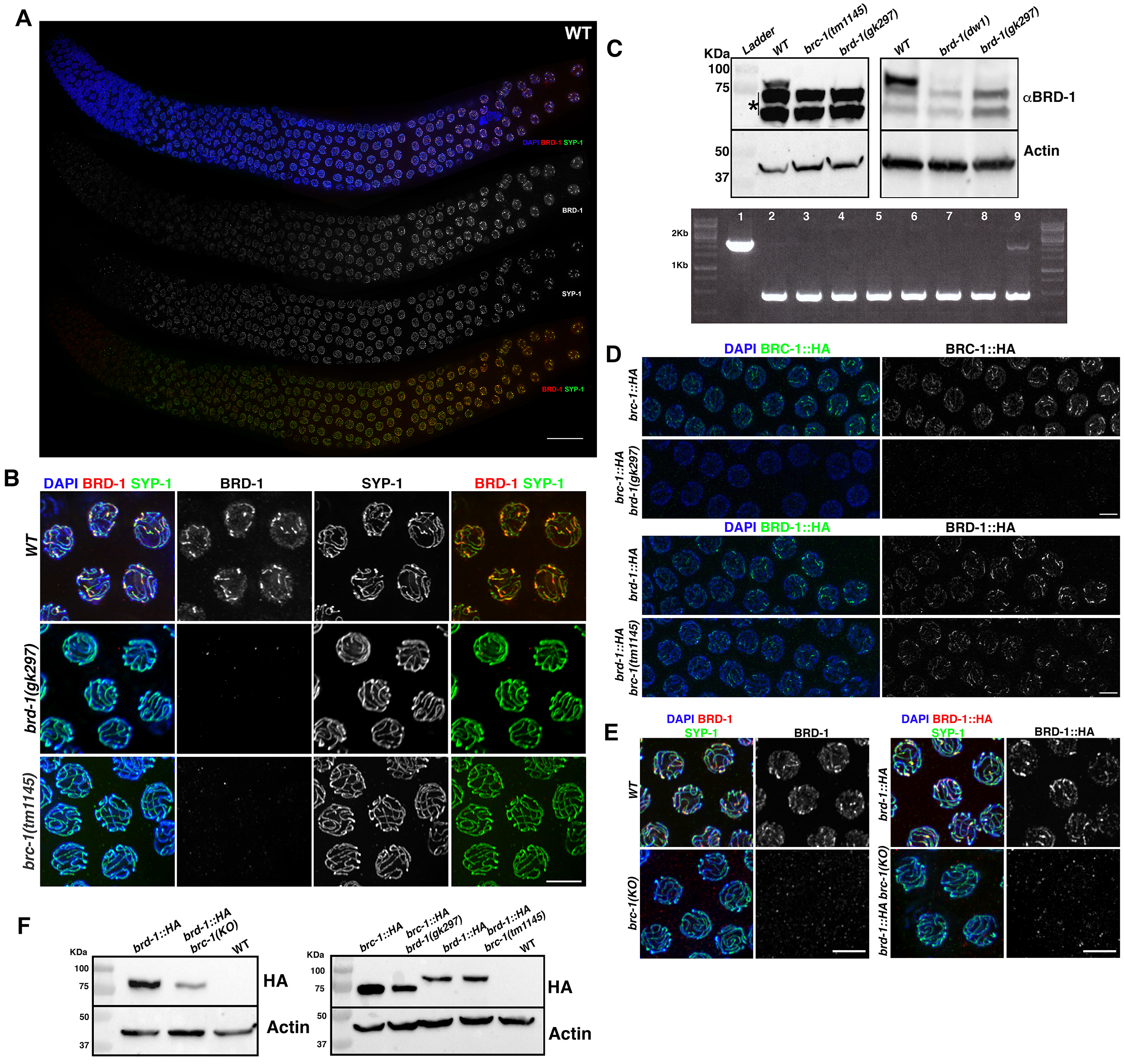

Supplement: S1 Fig — (A) Anti-BRD-1 and anti-SYP-1 immunostaining in wild-type animals shows that the BRD-1 expression pattern is identical to the one observed for BRC-1::HA. Note enrichment on the SC and retraction to the short arms of bivalents. Scale bar, 30 μm. (B) Late pachytene nuclei stained with anti BRD-1 and SYP-1 antibodies reveal lack of BRD-1 in brc-1(tm1145) mutants. Scale bar, 5 μm. (C) Left: western blot analysis on whole worm extracts shows that BRD-1 is not expressed in brc-1(tm1145) and that both brd-1(dw1) and brd-1(gk297) are null alleles of brd-1. Asterisk indicates a non-specific doublet recognised by the anti BRD-1 antibody. Right: genotyping for brd-1(dw1) reveals dw1 deletion in brc-1(tm1145) mutants and all the strains employed in this study. Numbers indicate different strains: 1-WT; 2-brc-1(tm1145), 3-brd-1(dw1), 4-brc-1(tm1145); msh-5/nT1, 5-brc-1(tm1145); [rpa-1::YFP], 6-brc-1(tm1145); GFP::msh-5, 7-brc-1(tm1145) OLLAS::cosa-1; GFP::rmh-1, 8-brc-1(tm1145); syp-2/nT1, 9-cosa-1(tm3298) brc-1(tmm145)/qC1. (D) Top: late pachytene nuclei stained with HA antibodies, showing that BRC-1::HA is not detected in brd-1(gk297). Bottom: BRD-1::HA is normally loaded in brc-1(tm1145) mutants. (E) Endogenous BRD-1 and BRD-1::HA are not loaded in brc-1(KO) knock outs, proving loading interdependency between BRC-1 and BRD-1. (F) Western blot analysis shows expression of BRD-1::HA and BRC-1::HA in the relevant genetic backgrounds. WT (N2) worms were used as negative controls and actin was used as loading control. Note that BRD-1::HA and BRC-1::HA displayed reduced levels in null brc-1(KO) and brd-1(gk297) mutants. (TIF) [file pgen.1007653.s001.tif]

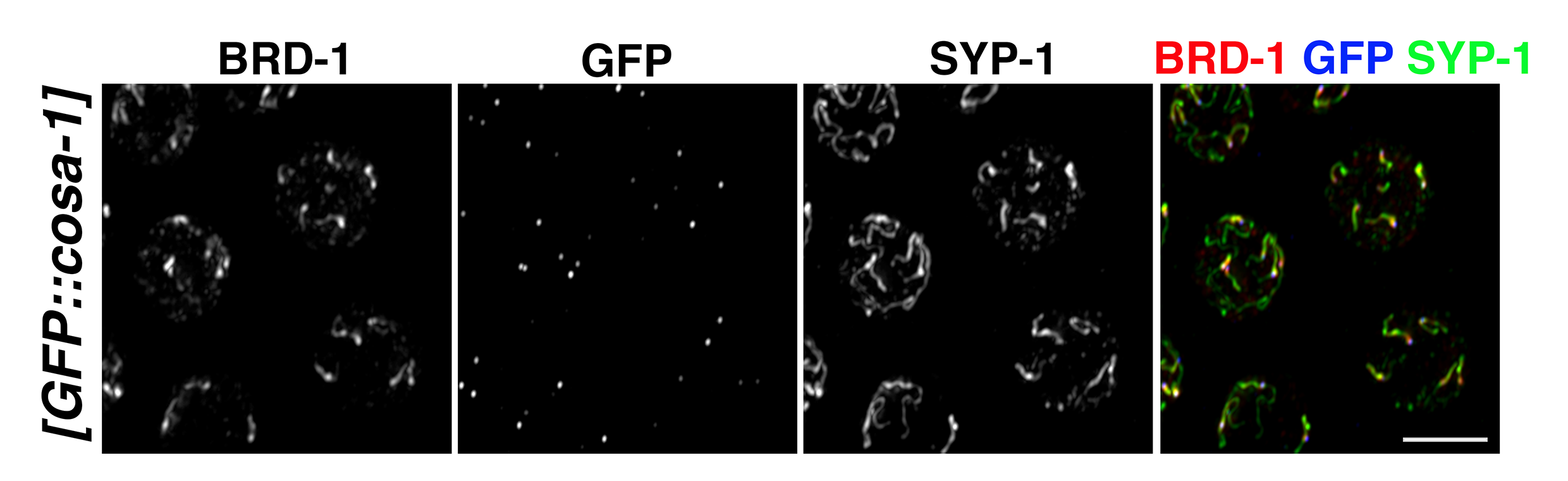

Supplement: S2 Fig — Late pachytene nuclei of [GFP::cosa-1] animals were stained for BRD-1, GFP and SYP-1. As previously observed for BRC-1::HA, BRD-1 is progressively enriched at the short arm of the bivalent, also containing COSA-1-labeled CO site. Scale bar, 5 μm. (TIF) [file pgen.1007653.s002.tif]

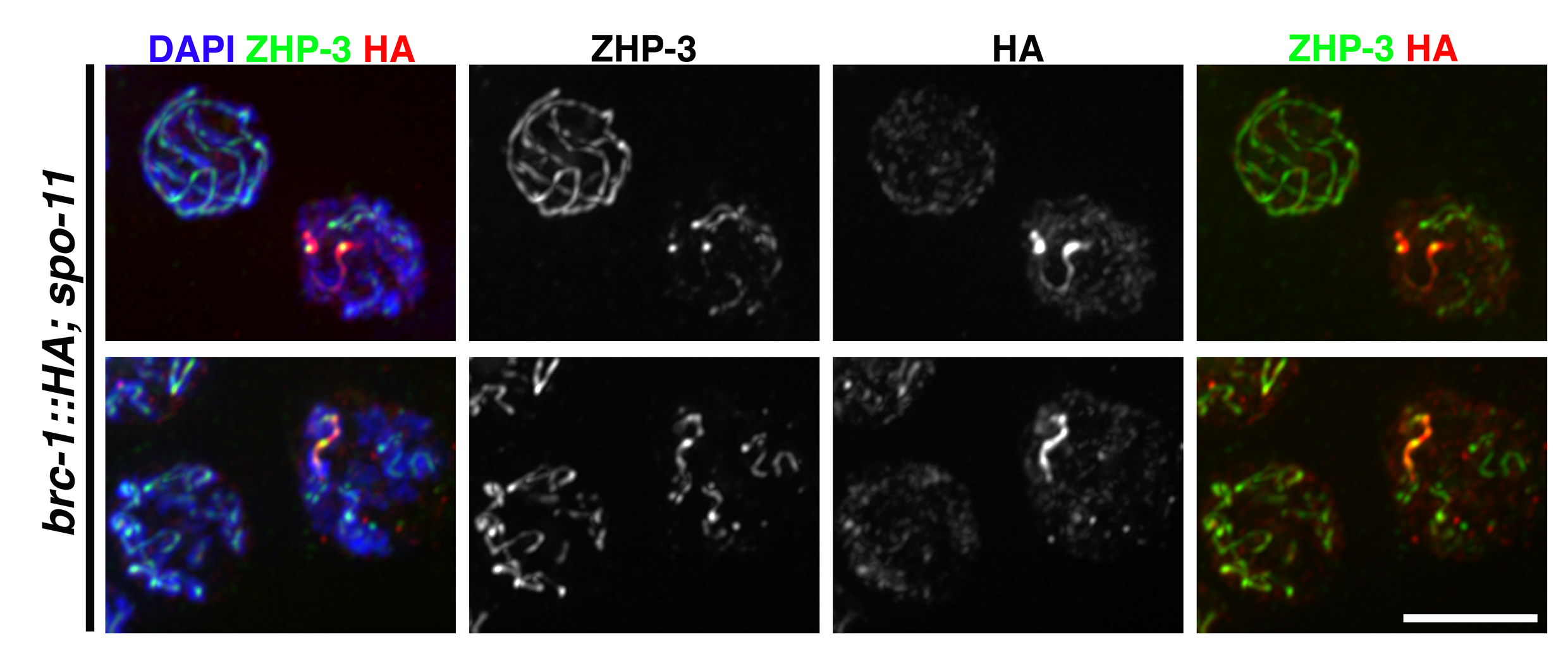

Supplement: S3 Fig — Two examples of late pachytene-diplotene nuclei in non-irradiated spo-11 mutants stained for BRC-1::HA and ZHP-3 showing retraction to the short arm of the bivalent. Scale bar, 5 μm. (TIF) [file pgen.1007653.s003.tif]

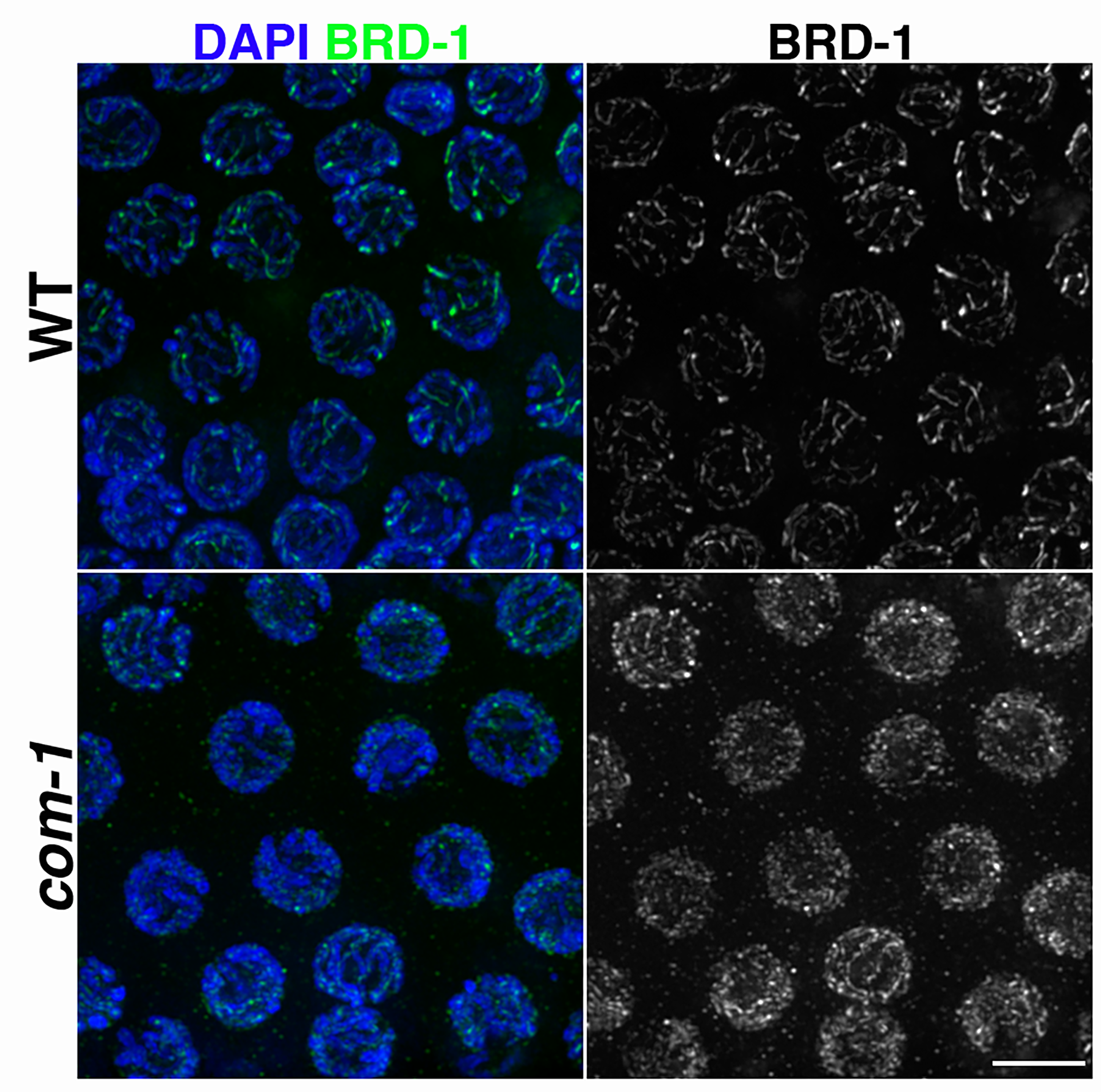

Supplement: S4 Fig — Mid-/late pachytene nuclei of the wild type (WT) and com-1 mutant were stained for BRD-1. BRD-1 loading onto the SC is drastically reduced when DNA resection is impaired. Scale bar, 5 μm. (TIF) [file pgen.1007653.s004.tif]

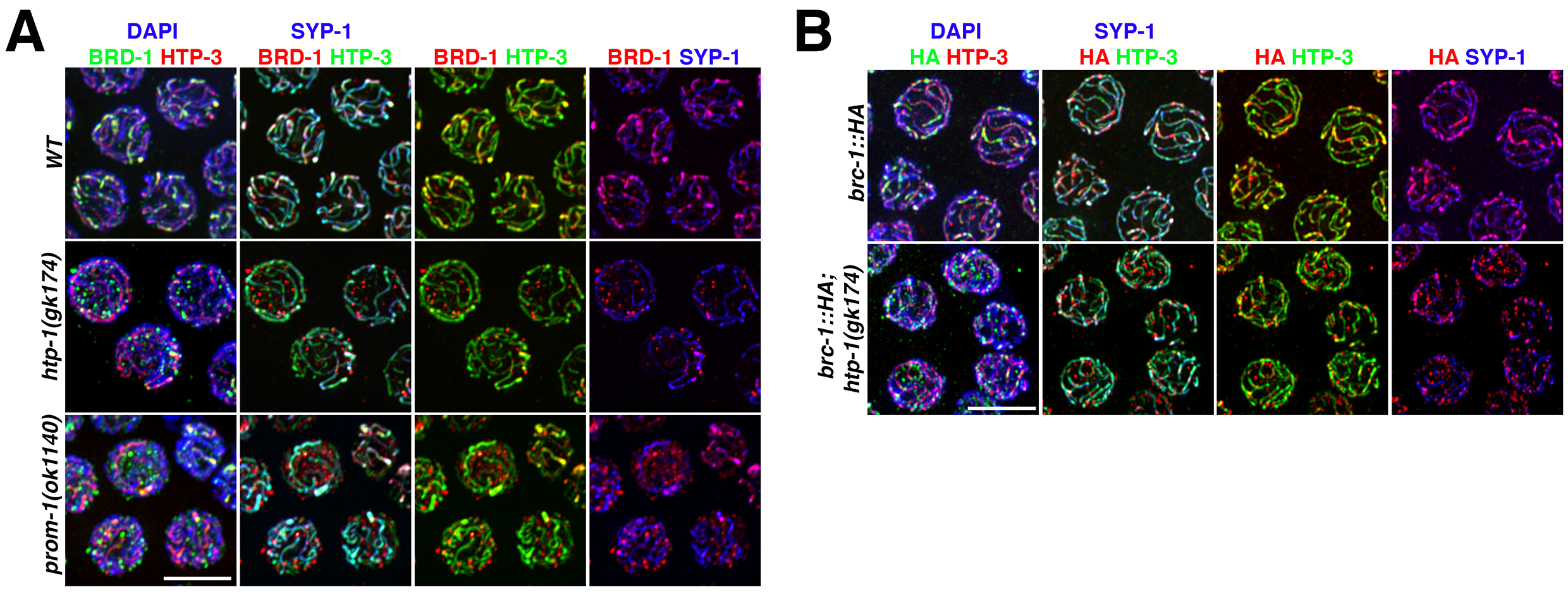

Supplement: S5 Fig — (A) Late pachytene nuclei in the wild-type (WT), htp-1 and prom-1 mutants were stained with BRD-1, SYP-1 and HTP-3. In both mutants, BRD-1 is largely excluded from the SC and forms nucleoplasmic agglomerates. (B) A similar staining pattern was observed for BRC-1::HA in htp-1 null mutants. Scale bar, 5 μm. (TIF) [file pgen.1007653.s005.tif]

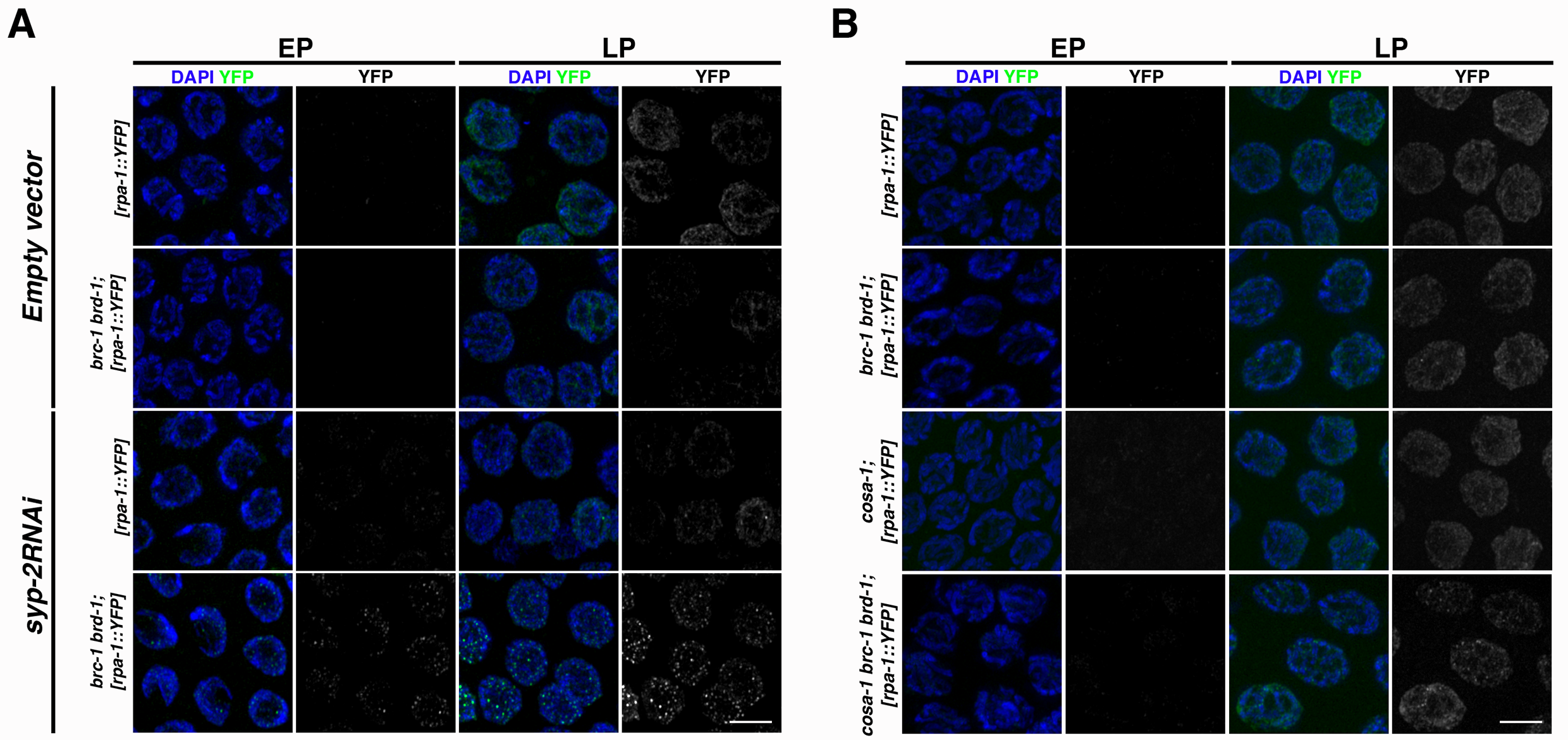

Supplement: S6 Fig — (A) Impairment of brc-1 brd-1 function upon synapsis deficiency causes accumulation of RPA-1::YFP in pachytene nuclei. EP = early pachynema, LP = late pachynema. (B) In cosa-1 brc-1 brd-1 mutants, dim RPA-1::YFP foci were only occasionally detected in few cells in late pachynema, suggesting that in absence of COs, impaired function of BCD complex in presence of functional SC does not prevent RPA-1/RAD-51 exchange. This is consistent with defective RAD-51 loading observed in brc-1 brd-1; syp-2 mutants but not in cosa-1 brc-1 brd-1. Scale bar, 5 μm. (TIF) [file pgen.1007653.s006.tif]

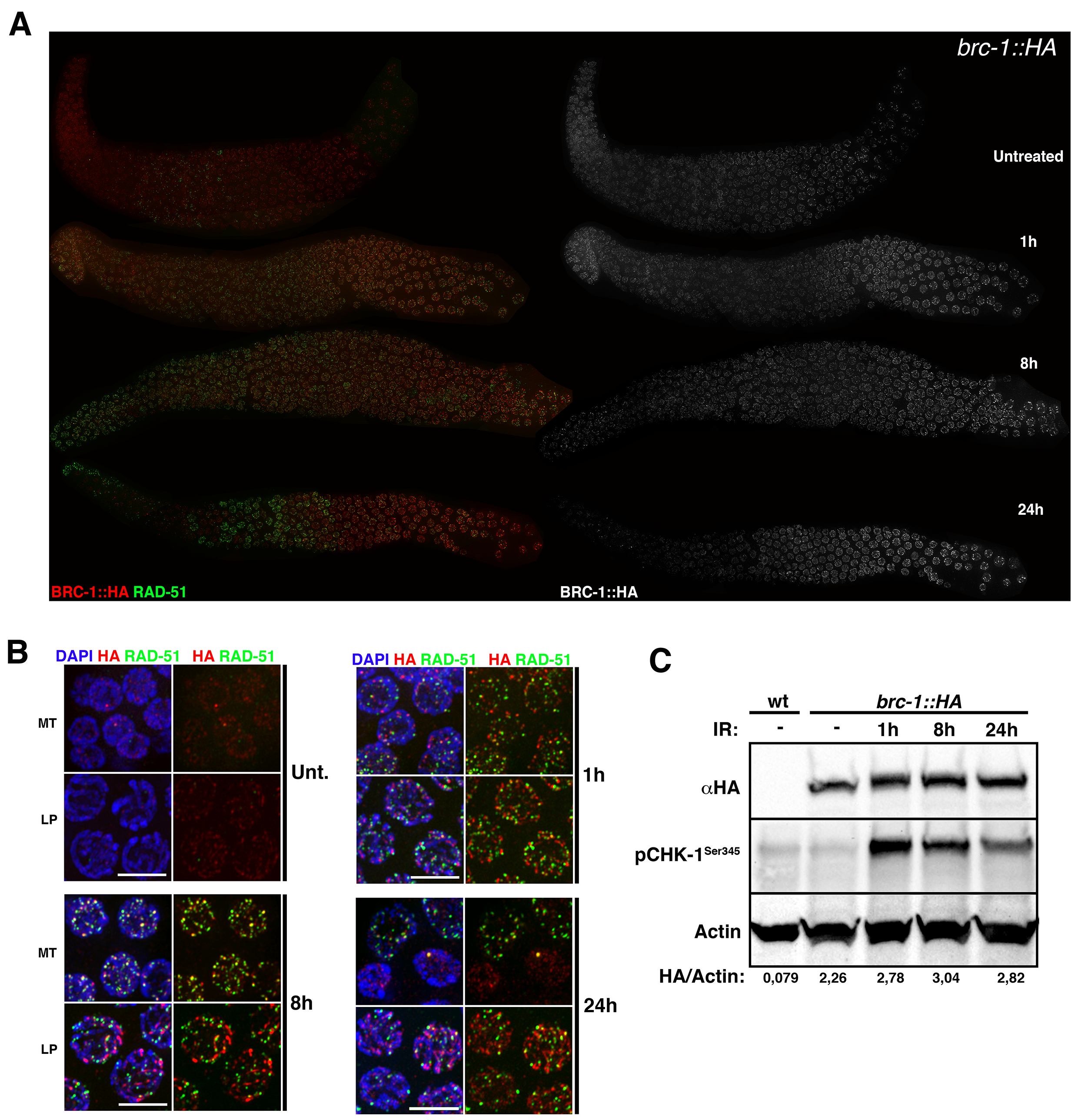

Supplement: S7 Fig — (A) Whole-mount gonads of irradiated and non-irradiated brc-1::HA worms immunostained for HA and RAD-51. Animals were exposed 75 Gy IR and analyzed at the indicated time points. (B) Representative nuclei from the pre-meiotic region (MT) and late pachytene (LP) stage of gonads analyzed at different times after IR. Note BRC-1::HA focus formation in pre-meiotic nuclei, along with robust co-localization with RAD-51 at 8 hours and occasionally at 24 hours post-irradiation. Scale bars, 5 μm. (C) Western blot analysis of whole-cell extracts shows a shift in BRC-1::HA migration after irradiation. Wild-type (WT) worms were used as negative control. Actin was the loading control and induction of phosphorylated CHK-1Ser345 was used as a positive control for irradiation. The ratio of BRC-1::HA to actin (HA/Actin) is shown as an abundance index. (TIF) [file pgen.1007653.s007.tif]
