# Supplementary material for: BRCA1-BARD1 associate with the synaptonemal complex and pro-crossover factors and influence RAD-51 dynamics during Caenorhabditis elegans meiosis
Source: PLoS Genet. 2018 Nov 1;14(11):e1007653. doi: 10.1371/journal.pgen.1007653 (PMC6211622; doi:10.1371/journal.pgen.1007653)
Supplement: S2 Table — T test was performed on RAD-51 foci number in different genotypes from transition zone to pachynema, corresponding to zone 4, 5, 6 and 7. (DOCX) [file pgen.1007653.s009.docx]

| **ZONE 4** | **p value** |
| --- | --- |
| *WT=msh-5* | 0,0015 |
| *WT=brc-1 brd-1; msh-5* | < 0,0001 |
| *brc-1= msh-5* | < 0,0001 |
| *brc-1= brc-1 brd-1; msh-5* | < 0,0001 |
| *msh-5= brc-1 brd-1; msh-5* | < 0,0001 |
| **ZONE 5** | **p value** |
| *WT=msh-5* | < 0,0001 |
| *WT=brc-1 brd-1; msh-5* | < 0,0001 |
| *brc-1= msh-5* | < 0,0001 |
| *brc-1= brc-1 brd-1; msh-5* | < 0,0001 |
| *msh-5= brc-1 brd-1; msh-5* | < 0,0001 |
| **ZONE 6** | **p value** |
| *WT=msh-5* | < 0,0001 |
| *WT=brc-1 brd-1; msh-5* | < 0,0001 |
| *brc-1= msh-5* | < 0,0001 |
| *brc-1= brc-1 brd-1; msh-5* | < 0,0001 |
| *msh-5= brc-1 brd-1; msh-5* | 0,3086 |
| **ZONE 7** | **p value** |
| *WT=msh-5* | 0,0015 |
| *WT=brc-1 brd-1; msh-5* | < 0,0001 |
| *brc-1= msh-5* | < 0,0001 |
| *brc-1= brc-1 brd-1; msh-5* | < 0,0001 |
| *msh-5= brc-1 brd-1; msh-5* | < 0,0001 |

**Table S2**
